# Fine Particulate Matter Components and Emergency Department Visits for Cardiovascular and Respiratory Diseases in the St. Louis, Missouri–Illinois, Metropolitan Area

**DOI:** 10.1289/ehp.1307776

**Published:** 2015-01-09

**Authors:** Stefanie Ebelt Sarnat, Andrea Winquist, James J. Schauer, Jay R. Turner, Jeremy A. Sarnat

**Affiliations:** 1 Department of Environmental Health, Emory University, Atlanta, Georgia, USA; 2 Civil and Environmental Engineering, University of Wisconsin-Madison, Madison, Wisconsin, USA; 3 Energy, Environmental and Chemical Engineering, Washington University in St. Louis, St. Louis, Missouri, USA

## Abstract

**Background:**

Given that fine particulate matter (≤ 2.5 μm; PM_2.5_) is a mixture of multiple components, it has been of high interest to identify its specific health-relevant physical and/or chemical features.

**Objectives:**

We conducted a time-series study of PM_2.5_ and cardiorespiratory emergency department (ED) visits in the St. Louis, Missouri–Illinois metropolitan area, using 2 years of daily PM_2.5_ and PM_2.5_ component measurements (including ions, carbon, particle-phase organic compounds, and elements) made at the St. Louis-Midwest Supersite, a monitoring site of the U.S. Environmental Protection Agency Supersites ambient air monitoring research program.

**Methods:**

Using Poisson generalized linear models, we assessed short-term associations between daily cardiorespiratory ED visit counts and daily levels of 24 selected pollutants. Associations were estimated for interquartile range changes in each pollutant. To allow comparison of relationships among multiple pollutants and outcomes with potentially different lag structures, we used 3-day unconstrained distributed lag models controlling for time trends and meteorology.

**Results:**

Considering results of our primary models, as well as sensitivity analyses and models assessing co-pollutant confounding, we observed robust associations of cardiovascular disease visits with 17α(H),21β(H)-hopane and congestive heart failure visits with elemental carbon. We also observed a robust association of respiratory disease visits with ozone. For asthma/wheeze, associations were strongest with ozone and nitrogen dioxide; observed associations of asthma/wheeze with PM_2.5_ and its components were attenuated in two-pollutant models with these gases. Differential measurement error due to differential patterns of spatiotemporal variability may have influenced patterns of observed associations across pollutants.

**Conclusions:**

Our findings add to the growing field examining the health effects of PM_2.5_ components. Combustion-related components of the pollutant mix showed particularly strong associations with cardiorespiratory ED visit outcomes.

**Citation:**

Sarnat SE, Winquist A, Schauer JJ, Turner JR, Sarnat JA. 2015. Fine particulate matter components and emergency department visits for cardiovascular and respiratory diseases in the St. Louis, Missouri–Illinois, metropolitan area. Environ Health Perspect 123:437–444; http://dx.doi.org/10.1289/ehp.1307776

## Introduction

Substantial epidemiologic evidence supports an association between ambient fine particulate (PM_2.5_; particulate matter with aerodynamic diameter ≤ 2.5 μm) air pollution and acute cardiorespiratory health effects [U.S. Environmental Protection Agency (EPA) 2009]. Given that PM_2.5_ is a mixture of multiple components, it has been of high interest to identify its specific health-relevant physical and/or chemical features to more effectively guide air pollution regulation (Dominici et al. 2010; National Research Council 2004). Recent reviews of the PM_2.5_ toxicological and epidemiological literature (Chen and Lippmann 2009; Kelly and Fussell 2012; Reiss et al. 2007; Rohr and Wyzga 2012) provide some indication of differential toxicity across PM components, with stronger evidence for health effects of carbon-related components [e.g., organic carbon (OC) and elemental carbon (EC)] and some metals [e.g., nickel, vanadium, zinc (Zn), lead (Pb)] than secondary inorganic components [e.g., sulfate (SO_4_^2–^) and nitrate (NO_3_^–^)]. However, studies have varied in their findings, perhaps for a number of factors, such as the specific components examined and differential measurement error among the components. Few epidemiological studies have assessed associations of health with specific organic PM species (Delfino et al. 2010; Kioumourtzoglou et al. 2013; Suh et al. 2011), partly because of the complexities in organics sampling and lack of available routine measurements.

For PM_2.5_ components that are measured routinely, a considerable limitation for many studies has been insufficient temporal resolution of PM component data. Routine measurements made by local and federal monitoring programs are generally available only every 3 or 6 days, which limits their usefulness for studies of associations between health outcomes and daily variations in pollutant concentrations. One approach to using these data has been hierarchical analyses that seek to determine whether associations with PM_2.5_ vary by average PM_2.5_ composition across geographic areas (Bell et al. 2009; Franklin et al. 2008; Zanobetti et al. 2009). Although these studies have provided important insight into possible composition-related effects of PM_2.5_, they have not been able to identify specific components as being associated with adverse health on a day-to-day basis (Ito et al. 2011). Recent studies have also applied the non-daily data directly as predictors in epidemiologic analyses, but the non-daily data lead to reduced power and limited ability to assess lag structures (Ito et al. 2011; Levy et al. 2012; Peng et al. 2009), which may be an important consideration depending on the specific outcomes and components of interest (Kim et al. 2012).

A growing number of time-series studies use daily PM component data from special monitoring campaigns, though few have published epidemiologic findings on a broad range of particle components (Mostofsky et al. 2012; Ostro et al. 2009). Here, we conducted a time-series study of PM_2.5_ and cardiorespiratory emergency department (ED) visits in the St. Louis, Missouri–Illinois (MO-IL) metropolitan area. For this project we used 2 years of daily PM_2.5_ and PM_2.5_ component measurements (including ions, carbon, particle-phase organic compounds, and elements) made at the St. Louis-Midwest Supersite. The St. Louis-Midwest Supersite was a monitoring site of the U.S. EPA Supersites ambient air monitoring research program, at which intensive measurements of fine particles were made during 2001–2003 for the broad goal of addressing scientific uncertainties associated with PM_2.5_.

## Methods

*ED visit data*. Computerized billing records were obtained from the Missouri Hospital Association (MHA) for emergency department visits to 36 of 43 acute care hospitals in the eight Missouri counties and eight Illinois counties of the St. Louis metropolitan statistical area (see Supplemental Material, Figure S1) for a 23-month study period (1 June 2001 through 30 May 2003) during which daily PM_2.5_ and PM_2.5_ component data were available from the Supersite. Relevant data elements included a unique longitudinal patient identifier (consisting of numbers with no true identifying information), admission date, admission source, admission type, primary and secondary *International Classification of Diseases, 9th Revision* (ICD-9) diagnosis codes, and ZIP code of patient residence. We used these data in accordance with our data use agreement with the MHA. The Emory University Institutional Review Board approved this study and granted an exemption from informed consent requirements, given the minimal risk nature of the study and the infeasibility of obtaining informed consent from individual patients for > 1.7 million billing records. Visits by patients living in ZIP codes outside of the 269 St. Louis ZIP codes were excluded.

The individual-level data were aggregated to daily counts for the following outcome groups, identified using primary ICD-9 codes: cardiovascular disease (CVD), which included visits for ischemic heart disease (codes 410–414), cardiac dysrhythmia (427), congestive heart failure (CHF) (428), and other CVD (433–437, 440, 443–445, 451–453); and respiratory disease (RD), which included visits for pneumonia (480–486), chronic obstructive pulmonary disease (491, 492, 496), asthma/wheeze (493, 786.07), and other RD (460–466, 477). Using the longitudinal patient identifier, multiple visits by the same patient for the same condition on the same day were counted as a single visit.

*Air quality data*. We obtained data for ozone (O_3_), carbon monoxide (CO), nitrogen dioxide (NO_2_), sulfur dioxide (SO_2_), and PM_2.5_ from all monitoring sites that operated during the study period from the U.S. EPA Air Quality System (AQS) (see Supplemental Material, Figure S1). Daily metrics of interest for the current analysis were created: 8-hr maximum O_3_, 1-hr maximum CO, 1-hr maximum NO_2_, 1-hr maximum SO_2_, and 24-hr average PM_2.5_. Meteorological data on temperature and relative humidity at the St. Louis Lambert International Airport were obtained from the National Climatic Data Center.

The St. Louis-Midwest Supersite, located approximately 3 km east of the city’s central business district and collocated with the Tudor Ave. AQS site, collected daily 24-hr filter-based PM_2.5_ samples and analyzed them for total mass, ions, carbon [via the Aerosol Characterization Experiments-Asia protocol (Schauer et al. 2003)], and 40 elements via energy-dispersive X-ray fluorescence (Bae et al. 2006; Lee et al. 2006). Filters were also analyzed for > 100 particle-phase nonpolar organic compounds via solvent extraction gas chromatography mass spectrometry (GCMS) and thermal desorption (TD)–GCMS (Sheesley et al. 2007).

To provide insight into the role of PM_2.5_ components in PM_2.5_ epidemiology while limiting the overall number of comparisons, we chose a subset of representative species *a priori* for inclusion in the analysis. We selected species that represented different chemical component classes, which may plausibly confer different toxicities based on different chemical properties (Suh et al. 2011). We selected ion (SO_4_^2–^, NO_3_^–^) and total carbon (OC and EC) measures. We also assessed eight representative organic compounds, chosen previously for detailed characterization and for which the data were determined to be statistically similar between the two measurement methods (Sheesley et al. 2007): *n*-Alkanes [*n*-octacosane (Oct), *n*-nonacosane (Non)], hopanes [17α(H),21β(H)-29-norhopane (Nor), 17α(H),21β(H)-hopane (Hop)], and polycyclic aromatic hydrocarbons (PAHs) {chrysene (Chry), benzo[*b+k*]fluoranthene (BbkF), benzo[*a*]pyrene (BaP), indeno[1,2,3-*cd*]pyrene (IcdP)}. For elements, we focused on metals and metalloids from major elemental groups for which the number of samples below the detection limit (BDL) was < 5%. Consideration was also given to species associated with health outcomes in previous studies (Chen and Lippmann 2009; Kelly and Fussell 2012; Rohr and Wyzga 2012). Selected components included: silicon (Si, metalloid, 0% BDL); potassium (K, alkali metal, 0% BDL); calcium (Ca, alkaline earth metal, 0% BDL); transition metals iron (Fe, 0% BDL), copper (Cu, 2.6% BDL), and Zn (0% BDL); and Pb (basic metal, 0.7% BDL). The transition metals vanadium and nickel, found to be associated with health outcomes in previous studies (Bell et al. 2009; Lippmann et al. 2006), were not considered because of their low concentrations (with > 80% BDL) in St. Louis. Overall, including the criteria pollutants, we evaluated 24 pollutants arising from various primary and secondary sources (Table 1).

**Table 1:** Characterization and data summary of selected pollutants measured at the St. Louis-Midwest Supersite/Tudor Ave. AQS monitoring location, 1 June 2001–30 April 2003.*^a^*

Pollutant	Abbreviation	Unit	Temporal metric	No. of days	Mean ± SD	Percent PM_2.5_^*b*^	Formation type	Dominant source at the St. Louis-Midwest supersite/Tudor Ave. site
Fine particles and components
Total PM_2.5_ mass	PM_2.5_	μg/m^3^	24-hr avg	683	18.0 ± 8.3	100	Both	Multiple sources
Major ions
Sulfate	SO_4_^2–^	μg/m^3^	24-hr avg	694	4.0 ± 3.1	22	Secondary	Secondary formation
Nitrate	NO_3_^–^	μg/m^3^	24-hr avg	679	2.2 ± 2	12	Secondary	Secondary formation
Carbon
Organic carbon	OC	μg/m^3^	24-hr avg	680	3.8 ± 1.9	21	Both	Multiple sources
Elemental carbon	EC	μg/m^3^	24-hr avg	666	0.8 ± 0.5	4	Primary	Mobile source^*c*^
*n*-Alkanes
*n*-Octacosane	Oct (nC_28_)	ng/m^3^	24-hr avg	668	1.15 ± 1.84	0.006	Primary	Point source^*c*^
*n*-Nonacosane	Non (nC_29_)	ng/m^3^	24-hr avg	679	2.57 ± 3.28	0.014	Primary	Point source^*c*^
Hopanes
17α(H),21β(H)-29-Norhopane	Nor (C_29_αβ)	ng/m^3^	24-hr avg	679	0.53 ± 0.43	0.003	Primary	Mobile source^*c*^
17α(H),21β(H)-Hopane	Hop (C_30_αβ)	ng/m^3^	24-hr avg	680	0.35 ± 0.27	0.002	Primary	Mobile source^*c*^
PAHs
Chrysene	Chry (C_18_H_12_)	ng/m^3^	24-hr avg	679	0.38 ± 0.4	0.002	Primary	Winter combustion^*c*^^,^^*d*^
Benzo[*b*+*k*]fluoranthene	BbkF (C_20_H_12_)	ng/m^3^	24-hr avg	681	0.64 ± 0.65	0.004	Primary	Point source^*c*^
Benzo[*a*]pyrene	BaP (C_20_H_12_)	ng/m^3^	24-hr avg	672	0.22 ± 0.42	0.001	Primary	Winter combustion^*c*^^,^^*d*^
Indeno[1,2,3-*cd*]pyrene	IcdP (C_22_H_12_)	ng/m^3^	24-hr avg	667	0.29 ± 0.35	0.002	Primary	Point source^*c*^
Metals and metalloids
Silicon (metalloid)	Si	ng/m^3^	24-hr avg	677	125.9 ± 214.8	0.70	Primary	Soil^*c*^
Potassium (alkali metal)	K	ng/m^3^	24-hr avg	677	72.8 ± 86.2	0.40	Primary	Area or nonlocal point source^*e*^
Calcium (alkaline earth metal)	Ca	ng/m^3^	24-hr avg	677	125.1 ± 88.8	0.70	Primary	Area or nonlocal point source^*e*^
Iron (transition metal)	Fe	ng/m^3^	24-hr avg	677	126.0 ± 99.6	0.70	Primary	Point source^*e*^; steel processing^*f*^
Copper (transition metal)	Cu	ng/m^3^	24-hr avg	677	24.2 ± 49.9	0.13	Primary	Point source; copper production^*f*^
Zinc (transition metal	Zn	ng/m^3^	24-hr avg	677	44.9 ± 74.4	0.25	Primary	Point source^*e*^; zinc smelting^*f*^
Lead (basic metal)	Pb	ng/m^3^	24-hr avg	677	19.3 ± 36.5	0.11	Primary	Point source^*e*^; lead smelting^*f*^
Criteria gases
Ozone	O_3_	ppb	8-hr max	679	36.2 ± 19.7	—	Secondary	Secondary formation
Carbon monoxide	CO	ppm	1-hr max	683	1.0 ± 0.7	—	Primary	Mobile source
Nitrogen dioxide	NO_2_	ppb	1-hr max	676	31.3 ± 9.3	—	Secondary	Mobile source
Sulfur dioxide	SO_2_	ppb	1-hr max	694	27.0 ± 37.7	—	Primary	Power plant
Abbreviations: avg, average; max, maximum. ^***a***^Measurements of PM_2.5_ and PM_2.5_ components made by St. Louis-Midwest Supersite instrumentation, and measurements of criteria gases (O_3_, CO, NO_2_, and SO_2_) made by Tudor Ave. AQS instrumentation; all descriptive statistics exclude data from 4 July 2001, 4 July 2002, and 5 July 2002. ^***b***^Mean percentage of total PM_2.5_ mass that each PM_2.5_ component represented over the study period. ^***c***^Jaeckels et al. (2007). ^***d***^For example, natural gas combustion due to residential heating. ^***e***^Snyder et al. (2009). ^*f*^Lee et al. (2006).								

*Analysis*. Data from all AQS monitoring sites were used for spatiotemporal characterization of pollutant concentrations in the study area. For epidemiologic analyses, data on pollutants of interest were obtained from the Supersite/Tudor Ave. monitoring location. This single location had two distinct sets of instrumentation: the St. Louis-Midwest Supersite instruments for PM_2.5_ and PM_2.5_ components and Tudor Ave. AQS instruments for gaseous pollutants.

We estimated short-term associations between daily cardiorespiratory ED visit counts and daily levels of the 24 selected pollutants using Poisson generalized linear models. To allow comparison of relationships among the multiple components and outcomes with potentially different lag structures, we used 3-day unconstrained distributed lag models of lags 0–2 (where lag 0 refers to the day of the ED visit, lag 1 refers to the day before the visit, and so on). Models included indicator variables to control for season (i.e., fall, winter, spring, and summer; in models for respiratory outcomes only), day of week, holidays, and a single indicator variable to account for one hospital not providing data after 26 April 2002. Models also controlled for time trends using cubic splines for day of visit with monthly knots, and temperature: using cubic splines for lag 0 maximum temperature with knots placed at the 25th and 75th percentiles, cubic terms for 1- to 2-day moving-average minimum temperature, and cubic terms for 0- to 2-day moving-average dew point temperature (Strickland et al. 2010). Three days (4 July 2001 and 4–5 July 2002) for which PM_2.5_ and specific PM component concentrations were impacted by fireworks displays at U.S. Independence Day celebrations were excluded from all analyses (e.g., mean PM_2.5_ and K concentrations at the St. Louis Supersite on these days were 5 and 199 times higher, respectively, than the average concentrations observed over the study period). Summary rate ratios (RRs) from the distributed lag models were calculated by summing the coefficients from the model for each lag and exponentiating the sum. RRs and 95% confidence intervals (CIs) were expressed per interquartile range (IQR) increase in each pollutant’s concentration. Statistical significance of epidemiologic associations was assessed at an alpha level of 0.10, and strength of associations was assessed relative to the estimated association for PM_2.5_ by outcome of interest.

In sensitivity analyses, we evaluated model misspecification and the potential for residual confounding by temporal factors by estimating associations with pollutant concentrations on the day after the emergency department visit (lag –1) given pollutant levels on the days of interest (Flanders et al. 2011). We also examined the sensitivity of our results to alternate model specifications, including alternate time trend control (cubic spline for day of visit with two knots per month and one knot every 2 months, respectively, instead of one knot per month), and alternate temperature control (indicator variables for each degree Celsius instead of a cubic spline for lag 0 maximum temperature). To assess the robustness of our results to lag structure, we examined 5-day distributed lag models (lags 0–4), with control for minimum and dew point temperature adjusted to include the moving average of lags 1–4 and 0–4, respectively. Finally, we evaluated the potential for confounding of selected single-pollutant results by co-pollutants using two-pollutant models; pollutants for testing in two-pollutant models were selected if they had a single-pollutant RR that was equal to or greater than the smallest statistically significant single-pollutant RR > 1 for the outcome of interest. When controlling for PM_2.5_ in models of the major PM_2.5_ components (i.e., those contributing ≥ 4% to total PM_2.5_), we considered both models that controlled for total PM_2.5_ and models that controlled for the noncomponent portion of total PM_2.5_ to avoid “double-counting” (Mostofsky et al. 2012). For these analyses, we assumed SO_4_^2–^ was in the form of ammonium sulfate [(NH_4_)_2_SO_4_], and calculated the non-sulfate portion of PM_2.5_ as PM_2.5_ – (SO_4_^2–^ × 132/96) (Luttmann-Gibson et al. 2014). Analyses were performed using SAS version 9.3 (SAS Institute Inc., Cary, NC).

## Results

*Data characterization*. Our ED visit database included information on 1,733,543 ED visits for all diagnoses. Data were from 28 Missouri hospitals and 8 Illinois hospitals, and represented an estimated 88% of all ED visits to hospitals in the study area during the study period. There were 69,679 visits (mean, 99.7 visits/day) for CVD and 186,449 visits (mean, 266.7 visits/day) for RD (see Supplemental Material, Table S1).

Summary statistics for all pollutants measured at the Supersite/Tudor Ave. monitoring location are presented in Table 1. The PM_2.5_ components providing the largest contributions to total PM_2.5_ included SO_4_^2–^ (22%), NO_3_^–^ (12%), OC (21%), and EC (4%). The selected organics and metals contributed little to PM_2.5_ (< 1%). Total PM_2.5_ was most strongly correlated with SO_4_^2–^ (*r* = 0.78) and OC (*r* = 0.76) (see Supplemental Material, Table S2). Among the PM_2.5_ components, correlations were generally strongest within chemical groupings: for example, OC with EC (*r* = 0.60), Oct with Non (*r* = 0.68), Nor with Hop (*r* = 0.86), and among the four PAHs (*r* ≥ 0.70).

To evaluate the representativeness of the Supersite/Tudor Ave. measurements for the St. Louis study area, we assessed partial correlations (i.e., correlations adjusted for all covariates included in epidemiologic models) between these data and available data at other sites (Table 2; CO and PM_2.5_ components were measured at only three other sites, thereby yielding only three comparisons in this analysis). Intersite correlations were strong (median *r* ≥ 0.84) for secondary pollutants (PM_2.5_, SO_4_^2–^, NO_3_^–^, and O_3_), suggestive of low spatiotemporal heterogeneity for these pollutants over the area covered by the available monitors. The correlation analysis also suggested low spatiotemporal heterogeneity of the metals Si (median *r* = 0.96) and K (median *r* = 0.71). Given that these metals are often associated with airborne dust and biomass burning sources (Table 1), which may be localized in certain areas, it is possible that the observed correlations are not representative of the broader study area if there were impacts from these sources away from monitor locations.

**Table 2:** Summary of intersite partial Pearson correlations between St. Louis-Midwest Supersite/Tudor Ave. AQS data and data from other monitoring locations for each pollutant, 1 June 2001–30 April 2003.*^a^*

Pollutants	No. of other sites	Median correlation (range)
Fine particles and components	
24-hr avg PM_2.5_	12^*b*^	0.88 (0.46–0.95)
24-hr avg PM_2.5_	3^*c*^	0.88 (0.76–0.95)
Major ions
24-hr avg SO_4_^2–^	3	0.90 (0.76–0.94)
24-hr avg NO_3_^–^	3	0.88 (0.75–0.90)
Carbon
24-hr avg OC	3	0.43 (0.38–0.69)
24-hr avg EC	3	0.47 (0.37–0.52)
Metals and metalloids
24-hr avg Si	3	0.96 (0.68–0.96)
24-hr avg K	3	0.71 (0.60–0.74)
24-hr avg Ca	3	0.35 (0.30–0.37)
24-hr avg Fe	3	0.54 (0.39–0.74)
24-hr avg Cu	3	0.03 (–0.09–0.05)
24-hr avg Zn	3	0.03 (–0.02–0.11)
24-hr avg Pb	3	0.08 (0.04–0.22)
Criteria gases
8-hr max O_3_	13^*d*^	0.85 (0.72–0.94)
1-hr max CO	3	0.62 (0.17–0.71)
1-hr max NO_2_	8	0.64 (0.27–0.70)
1-hr max SO_2_	10	–0.03 (–0.10–0.12)
Abbreviations: avg, average; max, maximum. ^***a***^These are partial correlations, computed as the correlations between residuals from linear models for each of the pollutants that included all of the covariates in our epidemiologic models; all correlations exclude data from 4 July 2001, 4 July 2002, and 5 July 2002. ^***b***^Includes data from all sites at which PM_2.5_ was measured. ^***c***^Includes data from a subset of PM_2.5_ sites at which PM_2.5_ components were also measured (every 3 or 6 days). ^***d***^Only two sites other than Tudor Ave. provided year-round O_3_ data; restricting the analysis to these sites produced similar results: median, 0.84 (range, 0.82–0.87).		

Median intersite correlations were moderate (median *r* = 0.35–0.64) for OC, EC, Ca, and Fe, as well as the gases CO and NO_2_ (Table 2). These moderate correlations are reflective of source contributions throughout the monitored area, as anticipated for traffic-related pollutants (e.g., EC, CO, NO_2_), for example. The relatively strong correlation (median *r* = 0.54) among sites for Fe is surprising given previous work apportioning this component in part to point sources (i.e., steel processing) local to the Supersite (Table 1). Intersite correlations (median *r* ≤ 0.08) were low for the primary pollutant SO_2_ and other metals (Cu, Zn, Pb) largely originating from local industrial point sources (Table 1).

Measurements of organic species were not available at additional sites during our study period; however, the likely source origins of these components at the Supersite have been characterized (Jaeckels et al. 2007) (Table 1). Given this characterization, we may anticipate low to moderate spatiotemporal heterogeneity for the hopanes, chrysene, and benzo[*a*]pyrene (nonlocal sources) and high spatiotemporal heterogeneity for the *n*-alkanes, benzo[*b+k*]fluoranthene, and indeno[1,2,3-*cd*]pyrene (local point sources).

*Associations of cardiovascular ED visits and ambient pollutants*. For cardiovascular outcomes, 3-day distributed lag associations with PM_2.5_ were all close to the null and not statistically significant (Table 3); the most positive association with PM_2.5_ was for CHF [RR = 1.015 (95% CI: 0.980, 1.051) per 11.1-μg/m^3^ increase]. Compared with associations with PM_2.5_ for each outcome, stronger and statistically significant positive associations at the 0.10 level were observed for CVD and CHF with OC and EC [e.g., for CHF–EC, RR = 1.042 (95% CI: 1.014, 1.070) per 0.42-μg/m^3^ increase], for CVD with Nor and Hop, and for CHF with Hop and Zn. Several other pollutants (e.g., for CHF with Nor, BbkF, IcdP, Ca, and O_3_) showed stronger associations than did PM_2.5_, but these were not statistically significant. Associations for other outcomes were generally close to the null with no statistically significant positive associations at the 0.10 level. Overall, of 96 tested relationships, we observed eight significant positive associations and five significant negative associations at the 0.10 level.

**Table 3:** Associations of cardiovascular ED visits and ambient pollutants in St. Louis, 1 June 2001–30 April 2003.*^a^*

Pollutant	IQR	CVD^*b*^RR (95% CI)	Ischemic heart disease RR (95% CI)	Dysrhythmia RR (95% CI)	Congestive heart failure RR (95% CI)
Fine particles and components
24-hr avg PM_2.5_	11.1 μg/m^3^	0.999 (0.981, 1.016)	1.005 (0.975, 1.036)	0.999 (0.961, 1.039)	1.015 (0.980, 1.051)
Major ions
24-hr avg SO_4_^2–^	3.2 μg/m^3^	1.000 (0.986, 1.014)	1.004 (0.980, 1.028)	1.007 (0.977, 1.038)	1.008 (0.980, 1.036)
24-hr avg NO_3_^–^	2.3 μg/m^3^	1.002 (0.981, 1.024)	1.020 (0.983, 1.058)	1.009 (0.963, 1.057)	1.007 (0.967, 1.050)
Carbon
24-hr avg OC	2.4 μg/m^3^	1.015 (0.997, 1.033)*	1.009 (0.979, 1.041)	1.002 (0.965, 1.042)	1.036 (1.001, 1.072)**
24-hr avg EC	0.42 μg/m^3^	1.016 (1.002, 1.030)**	1.003 (0.979, 1.028)	1.010 (0.980, 1.041)	1.042 (1.014, 1.070)**
*n*-Alkanes
24-hr avg Oct	0.77 ng/m^3^	1.001 (0.994, 1.007)	1.001 (0.989, 1.012)	0.988 (0.974, 1.002)*	1.008 (0.995, 1.020)
24-hr avg Non	1.98 ng/m^3^	0.998 (0.989, 1.007)	1.001 (0.985, 1.017)	0.987 (0.968, 1.007)	1.002 (0.985, 1.020)
Hopanes
24-hr avg Nor	0.43 ng/m^3^	1.013 (0.998, 1.028)*	1.021 (0.995, 1.047)	0.989 (0.958, 1.021)	1.023 (0.994, 1.052)
24-hr avg Hop	0.24 ng/m^3^	1.012 (1.000, 1.025)*	1.011 (0.989, 1.033)	1.003 (0.976, 1.030)	1.023 (0.999, 1.048)*
PAHs
24-hr avg Chry	0.39 ng/m^3^	1.005 (0.991, 1.020)	1.001 (0.976, 1.026)	1.002 (0.971, 1.034)	1.013 (0.985, 1.041)
24-hr avg BbkF	0.61 ng/m^3^	1.007 (0.993, 1.020)	1.003 (0.980, 1.027)	0.996 (0.967, 1.025)	1.021 (0.995, 1.047)
24-hr avg BaP	0.19 ng/m^3^	1.001 (0.994, 1.008)	0.999 (0.987, 1.010)	0.998 (0.983, 1.013)	1.007 (0.994, 1.020)
24-hr avg IcdP	0.27 ng/m^3^	1.006 (0.995, 1.018)	1.004 (0.985, 1.023)	1.001 (0.977, 1.024)	1.018 (0.996, 1.040)
Metals and metalloids
24-hr avg Si	70.2 ng/m^3^	0.995 (0.991, 0.999)**	0.993 (0.985, 1.000)**	0.994 (0.984, 1.004)	0.998 (0.990, 1.007)
24-hr avg K	35.5 ng/m^3^	0.996 (0.987, 1.006)	0.988 (0.972, 1.004)	0.997 (0.976, 1.018)	1.002 (0.983, 1.022)
24-hr avg Ca	86.0 ng/m^3^	0.994 (0.980, 1.009)	0.981 (0.957, 1.005)	0.996 (0.966, 1.028)	1.021 (0.993, 1.050)
24-hr avg Fe	85.9 ng/m^3^	0.989 (0.978, 1.001)*	0.977 (0.958, 0.996)**	0.996 (0.971, 1.021)	1.006 (0.984, 1.029)
24-hr avg Cu	21.9 ng/m^3^	1.001 (0.994, 1.008)	1.004 (0.992, 1.016)	0.999 (0.983, 1.015)	0.994 (0.980, 1.008)
24-hr avg Zn	37.9 ng/m^3^	1.005 (0.998, 1.013)	1.006 (0.993, 1.018)	0.994 (0.978, 1.010)	1.017 (1.003, 1.031)**
24-hr avg Pb	14.1 ng/m^3^	1.001 (0.995, 1.007)	0.999 (0.989, 1.010)	1.003 (0.990, 1.017)	0.997 (0.985, 1.009)
Criteria gases
8-hr max O_3_	28.3 ppb	0.990 (0.953, 1.027)	0.989 (0.928, 1.054)	1.001 (0.922, 1.087)	1.057 (0.982, 1.139)
1-hr max CO	0.7 ppm	1.005 (0.991, 1.018)	1.008 (0.985, 1.031)	1.003 (0.974, 1.032)	1.015 (0.989, 1.041)
1-hr max NO_2_	12.0 ppb	1.010 (0.990, 1.030)	1.018 (0.985, 1.053)	1.027 (0.984, 1.072)	1.011 (0.973, 1.050)
1-hr max SO_2_	24.0 ppb	1.006 (0.997, 1.015)	0.998 (0.982, 1.014)	1.016 (0.996, 1.037)	1.006 (0.988, 1.024)
Abbreviations: avg, average; max, maximum. ^***a***^All results from primary 3-day (lags 0–2) distributed lag models, with indicator variables to control for day-of-week, holidays, and to account for one hospital not providing data after 26 April 2002; cubic splines for day of visit with monthly knots; cubic spline for lag 0 maximum temperature with knots placed at the 25th and 75th percentiles; and cubic terms for 1- to 2-day moving-average minimum temperature and 0- to 2-day moving-average dew point temperature. ^***b***^CVD outcome included visits for ischemic heart disease (ICD-9 codes 410–414), cardiac dysrhythmia (ICD-9 code 427), congestive heart failure (ICD-9 code 428), and other CVD (ICD-9 codes 433–437, 440, 443–445, 451–453; i.e., peripheral and cerebrovascular diseases). *0.05 ≤ *p* < 0.10. **p < 0.05.					

Several sensitivity analyses were performed, and results of these analyses for CVD and CHF are presented in Supplemental Material, Tables S3–S6. Statistically significant associations with the following day’s pollutant levels (lag –1) given pollutant levels on the days of interest were observed with some pollutants (e.g., for CVD with OC, Oct, Non, and Nor, and for CHF with Nor, Hop, Fe, and O_3_); these lag –1 associations are assumed to reflect noncausal mechanisms of association because the exposures occurred after the outcome, suggesting the possibility of some model misspecification and/or residual confounding in primary models assessing the effects of these pollutants (Flanders et al. 2011). With respect to misspecification, models with more or less stringent time trend or temperature control did not meaningfully change the estimated primary model associations for these relationships (i.e., CVD with OC, Oct, Non, and Nor; and CHF with Nor, Hop, Fe, and O_3_) or others (see Supplemental Material, Tables S3 and S5). The observed results, however, were sensitive in two-pollutant models. For CVD, the significant positive single-pollutant associations with OC, EC, and Nor were substantially reduced when controlling for Hop [i.e., OC RR = 0.999 (95% CI: 0.974, 1.026); EC RR = 1.001 (95% CI: 0.981, 1.022); Nor RR = 1.002 (95% CI: 0.971, 1.033)], whereas the estimated single-pollutant association for Hop [RR = 1.012 (95% CI: 1.000, 1.025)] remained similar in two-pollutant models (see Supplemental Material, Table S4). For CHF, the single-pollutant associations with all selected pollutants were substantially reduced when controlling for EC, whereas associations between CHF and EC remained robust in two-pollutant models (see Supplemental Material, Table S6). The association of CHF with EC was similar whether adjusting for total PM_2.5_ or the non-EC portion of PM_2.5_.

*Associations of respiratory ED visits with ambient pollutants*. For respiratory outcomes, 3-day distributed lag associations with PM_2.5_ were close to the null except for a statistically significant positive association for asthma/wheeze [RR = 1.040 (95% CI: 1.009, 1.071) per 11.1-μg/m^3^ increase] (Table 4). Slightly weaker but statistically significant positive associations for asthma/wheeze were also observed with several PM_2.5_ components (SO_4_^2–^, OC, EC, Hop, and Ca); associations for asthma/wheeze with O_3_ and NO_2_ were stronger than with PM_2.5_. Among the other outcomes, we observed statistically significant positive associations for RD with O_3_, and for chronic obstructive pulmonary disease with several organic components (Oct, Nor, Chry, and BbkF). Of 96 tested relationships, we observed 13 significant positive associations and 2 significant negative associations at the 0.10 level.

**Table 4:** Associations of respiratory emergency department visits and ambient pollutants in St. Louis, 1 June 2001–30 April 2003.*^a^*

Pollutant	IQR	Respiratory disease^*b*^RR (95% CI)	Pneumonia RR (95% CI)	Chronic obstructive pulmonary disease RR (95% CI)	Asthma/wheeze RR (95% CI)
Fine particles and components
24-hr avg PM_2.5_	11.1 μg/m^3^	0.994 (0.979, 1.010)	0.977 (0.951, 1.004)	0.990 (0.946, 1.037)	1.040 (1.009, 1.071)**
Major ions
24-hr avg SO_4_^2–^	3.2 μg/m^3^	0.998 (0.986, 1.011)	0.990 (0.967, 1.014)	0.983 (0.946, 1.021)	1.029 (1.004, 1.055)**
24-hr avg NO_3_^–^	2.3 μg/m^3^	0.999 (0.982, 1.016)	0.991 (0.962, 1.021)	0.984 (0.933, 1.038)	1.011 (0.977, 1.046)
Carbon
24-hr avg OC	2.4 μg/m^3^	0.995 (0.980, 1.009)	0.982 (0.956, 1.009)	1.016 (0.971, 1.063)	1.029 (1.000, 1.060)*
24-hr avg EC	0.42 μg/m^3^	0.998 (0.987, 1.009)	0.982 (0.961, 1.004)	1.017 (0.982, 1.054)	1.020 (0.998, 1.044)*
*n*-Alkanes
24-hr avg Oct	0.77 ng/m^3^	0.999 (0.994, 1.005)	1.003 (0.994, 1.013)	1.017 (1.001, 1.033)**	1.003 (0.993, 1.013)
24-hr avg Non	1.98 ng/m^3^	1.000 (0.993, 1.008)	0.998 (0.984, 1.012)	1.015 (0.992, 1.039)	1.003 (0.989, 1.018)
Hopanes
24-hr avg Nor	0.43 ng/m^3^	0.993 (0.981, 1.005)	0.987 (0.966, 1.009)	1.037 (1.000, 1.077)*	1.011 (0.988, 1.036)
24-hr avg Hop	0.24 ng/m^3^	1.001 (0.991, 1.012)	0.994 (0.975, 1.013)	1.021 (0.989, 1.054)	1.027 (1.006, 1.047)**
PAHs
24-hr avg Chry	0.39 ng/m^3^	0.993 (0.982, 1.004)	0.983 (0.963, 1.004)	1.033 (0.996, 1.070)*	1.018 (0.995, 1.042)
24-hr avg BbkF	0.61 ng/m^3^	0.997 (0.986, 1.008)	0.984 (0.964, 1.004)	1.029 (0.995, 1.064)*	1.017 (0.996, 1.039)
24-hr avg BaP	0.19 ng/m^3^	1.001 (0.995, 1.006)	0.996 (0.986, 1.007)	1.013 (0.996, 1.029)	1.006 (0.996, 1.017)
24-hr avg IcdP	0.27 ng/m^3^	1.005 (0.996, 1.015)	0.998 (0.981, 1.016)	1.014 (0.986, 1.042)	1.013 (0.996, 1.031)
Metals and metalloids
24-hr avg Si	70.2 ng/m^3^	1.001 (0.997, 1.005)	1.005 (0.998, 1.012)	0.995 (0.982, 1.008)	1.002 (0.994, 1.010)
24-hr avg K	35.5 ng/m^3^	0.998 (0.989, 1.006)	1.000 (0.985, 1.015)	0.988 (0.964, 1.013)	1.012 (0.994, 1.029)
24-hr avg Ca	86.0 ng/m^3^	1.004 (0.993, 1.016)	1.001 (0.980, 1.023)	1.003 (0.966, 1.040)	1.024 (1.001, 1.048)**
24-hr avg Fe	85.9 ng/m^3^	1.001 (0.991, 1.011)	1.006 (0.989, 1.024)	0.985 (0.955, 1.016)	1.014 (0.994, 1.034)
24-hr avg Cu	21.9 ng/m^3^	0.997 (0.992, 1.003)	1.003 (0.992, 1.013)	1.001 (0.984, 1.018)	1.000 (0.989, 1.011)
24-hr avg Zn	37.9 ng/m^3^	0.991 (0.985, 0.997)**	0.996 (0.986, 1.007)	0.991 (0.972, 1.010)	0.993 (0.981, 1.006)
24-hr avg Pb	14.1 ng/m^3^	0.998 (0.993, 1.004)	1.001 (0.991, 1.011)	0.989 (0.973, 1.006)	1.002 (0.992, 1.013)
Criteria gases
8-hr max O_3_	28.3 ppb	1.052 (1.018, 1.087)**	1.041 (0.979, 1.106)	0.978 (0.886, 1.080)	1.067 (1.001, 1.137)**
1-hr max CO	0.7 ppm	0.998 (0.988, 1.009)	1.002 (0.983, 1.022)	1.015 (0.982, 1.049)	1.015 (0.993, 1.036)
1-hr max NO_2_	12.0 ppb	1.006 (0.990, 1.023)	1.005 (0.975, 1.036)	1.023 (0.973, 1.075)	1.050 (1.018, 1.084)**
1-hr max SO_2_	24.0 ppb	0.995 (0.988, 1.002)	0.992 (0.978, 1.005)	0.978 (0.956, 1.001)*	0.996 (0.981, 1.011)
Abbreviations: avg, average; max, maximum. ^***a***^All results from primary 3-day (lags 0–2) distributed lag models, with indicator variables to control for season, day-of-week, holidays, and to account for one hospital not providing data after 26 April 2002; cubic splines for day of visit with monthly knots; cubic spline for lag 0 maximum temperature with knots placed at the 25th and 75th percentiles; and cubic terms for 1- to 2-day moving-average minimum temperature and 0- to 2-day moving-average dew point temperature. ^***b***^RD outcome included visits for pneumonia (ICD-9 codes 480–486), chronic obstructive pulmonary disease (ICD-9 codes 491, 492, 496), asthma/wheeze (ICD-9 codes 493, 786.07), and other RD (ICD-9 codes 460–466, 477; i.e., upper respiratory infection and bronchiolitis). *0.05 ≤ *p* < 0.10. **p < 0.05.					

Sensitivity analysis results for asthma/wheeze are presented in Supplemental Material, Tables S7 and S8). Analyses of associations with the following day’s pollution levels (lag –1) given pollution levels on the days of interest suggested the possibility of some model misspecification and/or residual confounding in primary models for some pollutants (e.g., for asthma/wheeze, PM_2.5_, NO_3_^–^, and Cu each had significant lag –1 associations). Models with more or less stringent time trend or temperature control did not meaningfully change the lack of statistically significant positive associations with NO_3_^–^ or Cu in primary models. For PM_2.5_ and most other pollutants, associations for asthma/wheeze were sensitive to choice of time trend control (estimated associations from models with two knots per month were attenuated relative to our primary models with one knot per month). The 5-day distributed lag estimates for asthma/wheeze were generally stronger than the 3-day distributed lag estimates, and significant single-pollutant associations were noted for several additional components [IcdP: RR = 1.028 (95% CI: 1.004, 1.054); K: RR = 1.027 (95% CI: 1.003, 1.053); Fe: RR = 1.044 (95% CI: 1.017, 1.072) per IQR] that were not observed when evaluating 3-day distributed lag models; although the 3-day distributed lag estimates for these pollutants were not significant and were closer to the null than the 5-day distributed lag estimates, they were still positive, with RRs of 1.012 to 1.014 per IQR.

In two-pollutant models for asthma/wheeze, the significant single-pollutant associations for PM_2.5_ as well as those for SO_4_^2–^, OC, EC, Hop, and Ca were each substantially reduced (although remained largely positive) when controlling for either O_3_ or NO_2_, whereas associations with O_3_ and NO_2_ were largely stable on adjustment by co-pollutants and appeared the strongest of all pollutant associations (see Supplemental Material, Table S8). In two-pollutant models for RD, the single-pollutant association with O_3_ [RR = 1.052 (95% CI: 1.018, 1.087) per IQR] was not meaningfully altered by controlling for any pollutant examined here, with statistically significant RRs ranging from 1.048 to 1.077 after co-pollutant adjustment (results not shown).

**Figure 1:**
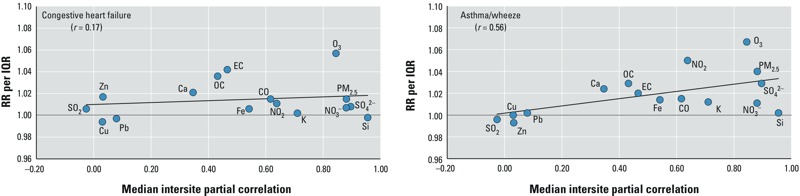
Relationships between median intersite partial pollutant correlations and rate ratios for congestive heart failure and asthma/wheeze ED visits. Trend lines indicate linear association between intersite correlations and RRs for each pollutant; *r*-value reflects Pearson correlation between intersite correlations and RRs; plots do not include results for alkanes, hopanes, or PAHs because these measures were only available at the Supersite/Tudor Ave. monitoring location so intersite correlations could not be computed.

*Relationships between pollutant spatiotemporal variability and rate ratios*. To provide some assessment of the potential for differential measurement error due to pollutant spatiotemporal variability to have affected the relative strengths of observed associations among the various pollutants, we examined the relationship between the median intersite partial correlations for pollutants measured at multiple monitoring sites (presented in Table 2) and the estimated RRs for CHF (presented in Table 3) and asthma/wheeze (presented in Table 4), including calculation of the Pearson correlation between the median intersite correlations and the RRs (Figure 1). The basis for this assessment was the idea that measurement error can lead to bias toward the null in estimated associations, with the bias potentially being greatest for pollutants with the most measurement error. For asthma/wheeze, the pollutants with strongest single-pollutant associations (i.e., PM_2.5_, O_3_, and NO_2_) had among the highest intersite correlations (*r* = 0.56), whereas for CHF there was little evidence for a relationship between the intersite correlations and the estimated strengths of pollutant associations (*r* = 0.17).

## Discussion

In this analysis, we assessed cardiovascular and respiratory ED visits in relation to daily levels of PM_2.5_ and PM_2.5_ components representing a range of chemical groups, including ions, carbons, particle-phase organics, and metals in St. Louis over a 23-month period. Relatively few time-series studies have considered such a broad range of daily measured particle components within a single study. Considering results of our primary models, as well as sensitivity analyses and models assessing co-pollutant confounding, we observed a robust association of CVD with 17α(H),21β(H)-hopane and CHF with EC. We also observed robust associations of RD with O_3_ and asthma/wheeze with O_3_ and NO_2_. Observed associations of asthma/wheeze with PM_2.5_ and its components were attenuated in two-pollutant models with these gases.

Of interest to us in evaluating the results of analyses assessing cardiovascular outcomes was whether the estimated associations were stronger for the PM_2.5_ mixture as a whole, or for specific PM_2.5_ components. Although the confidence intervals overlapped in all cases, we found trends of stronger associations with both major and minor PM_2.5_ components [EC and 17α(H),21β(H)-hopane] for CVD outcomes than for total PM_2.5_. These results are consistent with findings from a recent review of the literature (Rohr and Wyzga 2012). The observation that for CVD outcomes certain PM_2.5_ components showed stronger associations than total PM_2.5_ suggests that PM_2.5_ epidemiology may provide conservative estimates of health effects, depending on the contribution of these components.

Our results also concur with previous literature regarding specific components for which there is evidence of cardiovascular health effects, particularly carbon-related components OC and EC (Kelly and Fussell 2012; Rohr and Wyzga 2012). One plausible interpretation of our results may be that EC and 17α(H),21β(H)-hopane are themselves causally related to cardiovascular health end points. Alternatively, observed associations with these components may be indicative of a true causal agent within broader health-relevant mixtures from motor vehicle or other combustion sources; this may be especially true of 17α(H),21β(H)-hopane, given its very low contribution to total PM_2.5_ and OC mass. Differential particle size distribution of these specific components may also contribute to differences in estimated associations. OC and EC, for example, can account for up to 80–90% of ultrafine particle mass (Mauderly and Chow 2008), whereas other PM_2.5_ components (e.g., SO_4_^2–^) may occur predominantly in larger size fractions. Although carbonaceous components are frequently associated with cardiovascular outcomes in the literature, there is less consistency of associations with these components for respiratory outcomes (Rohr and Wyzga 2012). Here, we observed stronger associations of respiratory disease and asthma/wheeze ED visits with the gases O_3_ and NO_2_ than with PM_2.5_ or its components.

For studies, such as ours, that rely on a single central monitor to represent ambient pollutant concentrations over a large study area, a major consideration in comparing strengths of association among multiple components is the potential for different degrees of measurement error due to differences in spatial patterns of pollutant concentrations that influence the representativeness of central-site measurements. Pollutants with greater measurement error are likely to exhibit weaker associations with health outcomes than pollutants with less error, even if they are not inherently less toxic. This may be a particularly important consideration for our use of the Supersite/Tudor Ave. data, because this site was affected by local industrial sources, including a steel-processing facility, a copper-processing facility, a zinc smelter, and a lead smelter during the 2001–2003 study period (Lee et al. 2006; Maier et al. 2013). We assessed correlations of pollutant data available at multiple monitoring sites during the study period to provide an indication of spatiotemporal heterogeneity. Because of the limited spatial extent of monitoring sites in the study area (see Supplemental Material, Figure S1), this analysis provided only a rough assessment of pollutant spatial variability. These intersite correlations are likely affected by the number of monitors (e.g., only four monitors for PM_2.5_ components and CO), the distance between monitors, and monitor placement, which varied by pollutant.

For asthma/wheeze, the pollutants with strongest single-pollutant associations (i.e., PM_2.5_, O_3_, and NO_2_) had among the highest intersite correlations. In Figure 1, the positive association between single-pollutant RRs and median intersite partial correlations across pollutants suggests a downward bias of observed RRs for pollutants with higher spatiotemporal variability, which may be expected under a classical measurement error model (Peng and Bell 2010). These results suggest that different degrees of measurement error for different pollutants may have played a role in the observed patterns of associations across pollutants. For CHF, an examination of single-pollutant RRs in relation to the pollutant-specific median intersite partial correlations does not suggest influence of measurement error (due to spatiotemporal variability) on our findings, but this analysis had many limitations and thus it does not rule out such influence.

We limited our analysis to a subset of representative PM_2.5_ components detected and available from the St. Louis-Midwest Supersite. Although our findings are consistent overall with the existing literature, specific results may vary by study due to factors such as different pollutant mixtures, different degrees of measurement error for different pollutants, and/or different susceptibility of the populations. Kioumourtzoglou et al. (2013), for example, observed stronger associations of CVD hospital admissions with cyclohexanes than with hopanes in a three-city analysis including Atlanta, Georgia; Birmingham, Alabama; and Dallas, Texas. The 23-month time frame for our single-city study may have provided limited power to observe associations with pollutants for some outcomes and/or for certain PM components (Winquist et al. 2012). The specific time period analyzed, June 2001–May 2003, was based on availability of our highly unique speciated PM data from the St. Louis-Midwest Supersite, which did not make measurements on the full suite of PM components outside of this time frame. Pollutant concentrations around this monitoring location have changed over the last 10 years due to the shutdown of several nearby industrial point sources, likely reducing concentrations of specific metals assessed here, as well as the reduction in mobile source emissions as has occurred all around the United States. Although source strengths have changed over time, we anticipate that our observed component-specific effect estimates are relevant today and shed light on the potential health risk of commonly experienced pollutant mixtures.

A particular strength of this study was the availability of daily measurements of the multiple PM_2.5_ components. These data enabled evaluation of distributed lag models, as others have also recently done (Kim et al. 2012; Zhou et al. 2011), to allow comparison of relationships among the multiple components and outcomes with potentially different lag structures, as may be plausible due to different biological mechanisms. A study by Kim et al. (2012) found that associations with selected PM_2.5_ components were strongest at lag 0 for cardiovascular outcomes and at slightly longer lags for asthma. In the current study, the observation of stronger associations for asthma/wheeze when considering longer lags is consistent with these findings.

## Conclusions

Our study contributes new information to the growing, yet still limited body of research examining the health effects of PM_2.5_ components. Overall, we estimated positive associations of acute cardiovascular morbidity with carbon-containing PM [particularly EC and 17α(H),21β(H)-hopane] and of acute respiratory morbidity with O_3_ and NO_2_ in St. Louis.

## Supplemental Material

(2.4 MB) PDF
