# Supplementary material for: Fine Particulate Matter Components and Emergency Department Visits for Cardiovascular and Respiratory Diseases in the St. Louis, Missouri–Illinois, Metropolitan Area
Source: Environ Health Perspect. 2015 Jan 9;123(5):437–44. doi: 10.1289/ehp.1307776 (PMC4421761; doi:10.1289/ehp.1307776)
Supplement: (2.4 MB) PDF [file ehp.1307776.s001.508.pdf]

Environmental Health Perspectives doi:10.1289/ehp.1307776

Supplemental Material

Fine Particulate Matter Components and Emergency Department Visits for Cardiovascular and Respiratory Diseases in the St. Louis, Missouri–Illinois, Metropolitan Area

Stefanie Ebelt Sarnat, Andrea Winquist, James J. Schauer, Jay R. Turner, and Jeremy A. Sarnat

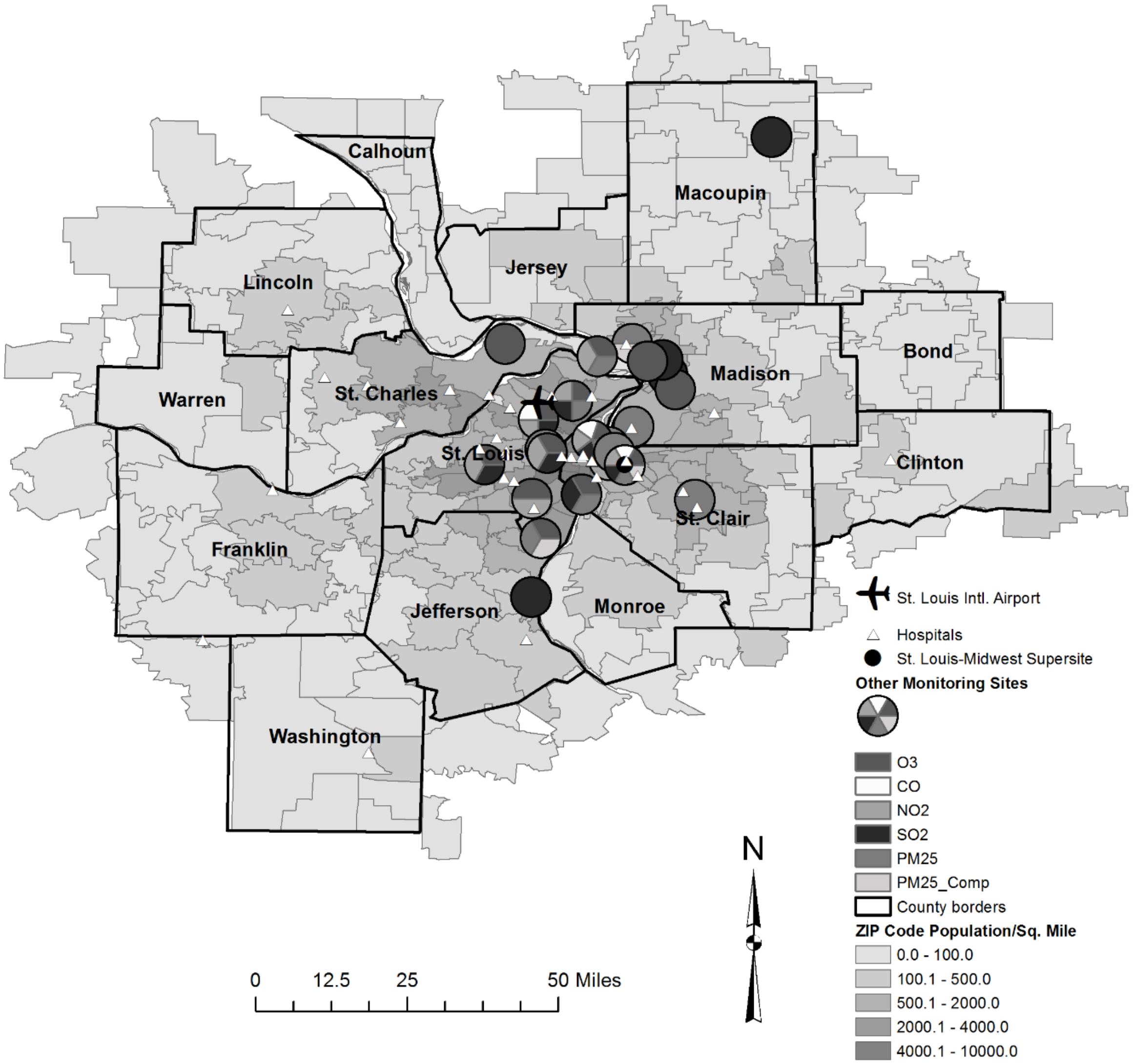

Figure S1. Map of St. Louis study area, with locations of hospitals and air quality monitoring sites.

Table S1. Emergency department visit summary statistics for St. Louis, 6/1/2001-4/30/2003.[a]

Outcome [ICD-9 Codes]	Total number of visits	Mean daily visits ± SD
**All Diseases**	**1,733,543**	**2,480.0 ± 160.9**
**Cardiovascular disease[b]**	**69,679**	**99.7 ± 12.8**
Ischemic heart disease [410-414]	22,097	31.6 ± 6.7
Dysrhythmia [427]	13,822	19.8 ± 4.8
Congestive heart failure [428]	17,148	24.5 ± 5.7
**Respiratory disease[c]**	**186,449**	**266.7 ± 72.0**
Pneumonia [480-486]	32,166	46.0 ± 14.5
Chronic obstructive pulmonary disease [491, 492, 496]	10,377	14.8 ± 4.6
Asthma or wheeze [493, 786.07]	34,086	48.8 ± 14.6

[a]Analyses exclude data from 7/4/2001, 7/4/2002, and 7/5/2002. [b]Cardiovascular disease outcome included visits for ischemic heart disease [410-414], cardiac dysrhythmia [427], congestive heart failure [428], and other CVD [433-437, 440, 443-445, 451-453; i.e., peripheral and cerebrovascular diseases]. [c]Respiratory disease outcome included visits for pneumonia [480-486], chronic obstructive pulmonary disease [491, 492, 496], asthma/wheeze [493, 786.07], and other RD [460-466, 477; i.e., upper respiratory infection and bronchiolitis].

Table S2. Pearson correlation coefficients among all pollutants measured at the St. Louis-Midwest Supersite/Tudor Ave. AQS monitoring location, 6/1/2001-4/30/2003.[a]

**Pollutant**	**$PM_{2.5}$**	**SO_4^{2-}**	**NO_3^-**	**OC**	**EC**	**Oct**	**Non**	**Nor**	**Hop**	**Chry**	**BbkF**	**BaP**	**IcdP**	**Si**	**K**	**Ca**	**Fe**	**Cu**	**Zn**	**Pb**	**O_3**	**CO**	**NO_2**	**SO_2**
**Fine Particles**																								
24-hr avg $PM_{2.5}$	1.00																							
Major Ions																								
24-hr avg SO_4^{2-}	0.78	1.00																						
24-hr avg NO_3^-	0.29	0.01	1.00																					
Carbon																								
24-hr avg OC	0.76	0.45	0.05	1.00																				
24-hr avg EC	0.33	0.00	0.12	0.60	1.00																			
n-Alkanes																								
24-hr avg Oct	0.32	0.05	0.11	0.50	0.51	1.00																		
24-hr avg Non	0.31	0.13	-0.06	0.51	0.40	0.68	1.00																	
Hopanes																								
24-hr avg Nor	0.25	-0.03	0.13	0.46	0.56	0.52	0.23	1.00																
24-hr avg Hop	0.30	0.01	0.17	0.55	0.58	0.53	0.35	0.86	1.00															
PAHs																								
24-hr avg Chry	0.05	-0.34	0.46	0.14	0.43	0.32	0.06	0.49	0.43	1.00														
24-hr avg BbkF	0.18	-0.20	0.39	0.28	0.54	0.43	0.17	0.55	0.47	0.89	1.00													
24-hr avg BaP	0.12	-0.24	0.26	0.30	0.60	0.45	0.27	0.51	0.46	0.77	0.86	1.00												
24-hr avg IcdP	0.20	-0.11	0.26	0.32	0.49	0.42	0.16	0.53	0.41	0.72	0.79	0.70	1.00											
Metals and Metalloids																								
24-hr avg Si	0.33	0.28	-0.30	0.45	0.26	0.23	0.38	0.17	0.24	-0.07	-0.03	0.02	0.04	1.00										
24-hr avg K	0.59	0.33	0.02	0.65	0.36	0.35	0.39	0.32	0.36	0.17	0.24	0.22	0.29	0.63	1.00									
24-hr avg Ca	0.35	0.12	-0.15	0.56	0.50	0.37	0.37	0.40	0.45	0.21	0.24	0.30	0.25	0.73	0.58	1.00								
24-hr avg Fe	0.46	0.27	-0.03	0.52	0.55	0.40	0.38	0.35	0.38	0.29	0.42	0.43	0.40	0.55	0.47	0.61	1.00							
24-hr avg Cu	0.16	0.08	-0.07	0.26	0.20	0.13	0.13	0.25	0.28	0.05	0.07	0.10	0.10	0.22	0.25	0.28	0.25	1.00						
24-hr avg Zn	0.34	0.11	0.14	0.35	0.36	0.20	0.16	0.30	0.27	0.29	0.34	0.32	0.37	0.23	0.37	0.40	0.48	0.47	1.00					
24-hr avg Pb	0.35	0.12	0.03	0.48	0.47	0.34	0.26	0.44	0.39	0.32	0.41	0.39	0.45	0.26	0.43	0.42	0.47	0.53	0.68	1.00				
**Criteria Gases**																								
8-hr max O_3	0.23	0.49	-0.57	0.30	-0.09	0.04	0.16	-0.07	-0.05	-0.42	-0.31	-0.27	-0.19	0.50	0.24	0.33	0.24	0.06	0.01	0.12	1.00			
1-hr max CO	0.25	-0.03	0.12	0.44	0.55	0.48	0.24	0.62	0.56	0.47	0.58	0.57	0.56	0.15	0.25	0.38	0.52	0.19	0.36	0.44	-0.01	1.00		
1-hr max NO_2	0.35	0.23	-0.06	0.51	0.44	0.36	0.21	0.52	0.48	0.24	0.30	0.30	0.33	0.36	0.37	0.50	0.44	0.18	0.27	0.38	0.37	0.54	1.00	
1-hr max SO_2	0.08	-0.05	0.05	0.10	0.07	0.02	-0.01	0.19	0.14	0.14	0.09	0.09	0.13	0.09	0.21	0.19	0.04	0.36	0.43	0.39	-0.04	0.12	0.15	1.00

[a]Measurements of $PM_{2.5}$ and $PM_{2.5}$ components made by St. Louis-Midwest Supersite instrumentation, and measurements of criteria gases (O_3, CO, NO_2, and SO_2) made by US Environmental Protection Agency Air Quality System Tudor Ave. instrumentation; all correlations exclude data from 7/4/2001, 7/4/2002, and 7/5/2002.

Table S3. Sensitivity analyses of associations between cardiovascular disease emergency department visits and ambient pollutants in St. Louis, 6/1/2001-4/30/2003.[a]

Pollutant	IQR	Primary Model[b]	Lag -1 Pollution[c]	2 Knots per Month[d]	1 Knot Every 2 Months[e]	Indicator Lag 0 Maximum Temp[f]	5-day Distributed Lag[g]
**Fine Particles and Components**							
24-hr avg $PM_{2.5}$	11.1 μg/m^3	0.999 (0.981, 1.016)	1.012 (0.996, 1.028)	0.994 (0.975, 1.013)	1.003 (0.987, 1.020)	0.999 (0.982, 1.017)	1.014 (0.992, 1.037)
Major Ions							
24-hr avg SO_4^{2-}	3.2 μg/m^3	1.000 (0.986, 1.014)	1.008 (0.994, 1.022)	0.999 (0.984, 1.014)	1.003 (0.989, 1.017)	0.999 (0.985, 1.013)	1.016 (0.998, 1.033)*
24-hr avg NO_3^-	2.3 μg/m^3	1.002 (0.981, 1.024)	1.007 (0.992, 1.022)	0.999 (0.976, 1.022)	1.006 (0.986, 1.026)	1.005 (0.983, 1.027)	1.018 (0.991, 1.046)
Carbon							
24-hr avg OC	2.4 μg/m^3	1.015 (0.997, 1.033)*	1.012 (0.999, 1.024)*	1.015 (0.996, 1.034)	1.016 (0.999, 1.033)*	1.021 (1.002, 1.040)**	1.031 (1.008, 1.055)**
24-hr avg EC	0.42 μg/m^3	1.016 (1.002, 1.030)**	1.000 (0.989, 1.010)	1.014 (0.999, 1.030)*	1.017 (1.003, 1.030)**	1.016 (1.002, 1.031)**	1.010 (0.991, 1.030)
n-Alkanes							
24-hr avg Oct	0.77 ng/m^3	1.001 (0.994, 1.007)	1.007 (1.001, 1.013)**	1.002 (0.994, 1.009)	1.002 (0.996, 1.008)	1.002 (0.995, 1.008)	0.999 (0.990, 1.009)
24-hr avg Non	1.98 ng/m^3	0.998 (0.989, 1.007)	1.007 (1.000, 1.013)**	0.995 (0.984, 1.007)	0.999 (0.991, 1.007)	1.000 (0.991, 1.009)	0.999 (0.988, 1.010)
Hopanes							
24-hr avg Nor	0.43 ng/m^3	1.013 (0.998, 1.028)*	1.009 (0.999, 1.019)*	1.014 (0.999, 1.030)*	1.013 (0.999, 1.028)*	1.015 (1.000, 1.030)*	1.015 (0.995, 1.036)
24-hr avg Hop	0.24 ng/m^3	1.012 (1.000, 1.025)*	1.007 (0.998, 1.015)	1.013 (1.000, 1.027)*	1.012 (1.000, 1.024)*	1.015 (1.002, 1.028)**	1.012 (0.994, 1.030)
PAHs							
24-hr avg Chry	0.39 ng/m^3	1.005 (0.991, 1.020)	1.004 (0.994, 1.015)	1.006 (0.991, 1.022)	1.008 (0.994, 1.022)	1.007 (0.993, 1.022)	1.001 (0.981, 1.021)
24-hr avg BbkF	0.61 ng/m^3	1.007 (0.993, 1.020)	1.004 (0.994, 1.014)	1.007 (0.992, 1.021)	1.010 (0.997, 1.023)	1.009 (0.995, 1.023)	1.005 (0.986, 1.024)
24-hr avg BaP	0.19 ng/m^3	1.001 (0.994, 1.008)	1.003 (0.999, 1.008)	0.998 (0.990, 1.006)	1.002 (0.996, 1.008)	1.000 (0.994, 1.007)	0.999 (0.990, 1.008)
24-hr avg IcdP	0.27 ng/m^3	1.006 (0.995, 1.018)	1.003 (0.995, 1.010)	1.005 (0.992, 1.017)	1.008 (0.997, 1.018)	1.006 (0.995, 1.017)	1.006 (0.991, 1.021)
Metals and Metalloids							
24-hr avg Si	70.2 ng/m^3	0.995 (0.991, 0.999)**	1.002 (0.997, 1.007)	0.993 (0.988, 0.998)**	0.996 (0.992, 1.000)*	0.995 (0.991, 1.000)**	0.994 (0.985, 1.004)
24-hr avg K	35.5 ng/m^3	0.996 (0.987, 1.006)	1.005 (0.995, 1.015)	0.995 (0.985, 1.005)	0.999 (0.991, 1.008)	0.997 (0.988, 1.007)	0.989 (0.975, 1.003)
24-hr avg Ca	86.0 ng/m^3	0.994 (0.980, 1.009)	1.001 (0.992, 1.011)	0.993 (0.978, 1.009)	0.993 (0.980, 1.007)	0.994 (0.980, 1.009)	0.993 (0.975, 1.011)
24-hr avg Fe	85.9 ng/m^3	0.989 (0.978, 1.001)*	1.001 (0.992, 1.011)	0.986 (0.974, 0.999)**	0.992 (0.981, 1.002)	0.991 (0.979, 1.002)	0.992 (0.976, 1.008)
24-hr avg Cu	21.9 ng/m^3	1.001 (0.994, 1.008)	1.002 (0.998, 1.006)	0.999 (0.992, 1.007)	1.000 (0.993, 1.007)	1.000 (0.993, 1.007)	1.000 (0.990, 1.009)
24-hr avg Zn	37.9 ng/m^3	1.005 (0.998, 1.013)	1.003 (0.998, 1.007)	1.002 (0.994, 1.010)	1.005 (0.998, 1.012)	1.005 (0.997, 1.012)	1.007 (0.997, 1.017)
24-hr avg Pb	14.1 ng/m^3	1.001 (0.995, 1.007)	1.002 (0.999, 1.005)	0.999 (0.992, 1.006)	1.001 (0.995, 1.007)	1.000 (0.994, 1.006)	1.001 (0.993, 1.010)
**Criteria Pollutants**							
8-hr max O_3	28.3 ppb	0.990 (0.953, 1.027)	1.001 (0.978, 1.024)	0.998 (0.958, 1.039)	0.994 (0.959, 1.030)	0.996 (0.959, 1.035)	0.991 (0.949, 1.034)
1-hr max CO	0.7 ppm	1.005 (0.991, 1.018)	1.003 (0.994, 1.013)	1.005 (0.990, 1.020)	1.005 (0.993, 1.018)	1.006 (0.992, 1.020)	1.005 (0.988, 1.022)
1-hr max NO_2	12.0 ppb	1.010 (0.990, 1.030)	1.007 (0.994, 1.021)	1.007 (0.985, 1.028)	1.012 (0.994, 1.030)	1.014 (0.994, 1.034)	1.013 (0.989, 1.038)
1-hr max SO_2	24.0 ppb	1.006 (0.997, 1.015)	0.999 (0.993, 1.005)	1.004 (0.993, 1.015)	1.005 (0.996, 1.014)	1.004 (0.994, 1.013)	1.005 (0.993, 1.018)

[a]Results provided as rate ratios (95% CI) per interquartile range increase in pollution. [b]"Primary Model" was a 3-day (lags 0-2) distributed lag model with: indicator variables to control for day-of-week, holidays, and to account for one hospital not providing data after April 26, 2002; cubic splines for day of visit with monthly knots; cubic spline for lag 0 maximum temperature with knots placed at the 25th and 75th percentiles; and cubic terms for 1-2 day moving average minimum temperature and 0-2 day moving average dew point temperature. [c]"Lag -1 Pollution" model was the same as the primary model, but included pollutant concentrations on the day after the emergency department visit (lag -1) given pollutant levels on the days of interest (results for lag -1 RRs reported here). [d]"2 Knots per Month" model was the same as the primary model, but included a cubic spline for day of visit with two knots instead of one knot per month. [e]"1 Knot Every 2 Months" model was the same as the primary model, but included a cubic spline for day of visit with one knot every two months instead of one knot every month. [f]"Indicator Lag 0 Max Temp" model was the same as the primary model, but included indicator variables for each degree Celsius of lag 0 max temp instead of a cubic spline. [g]"5-Day Distributed Lag" model was similar to the primary model, but examined pollutant lags 0-4 with control for minimum and dew point temperature adjusted to include the moving average of lags 1-4 and 0-4 respectively. ** = results with p-value<0.05; * = results with 0.05≤p-value<0.10.

Table S4. Comparison of selected single-pollutant model results for cardiovascular disease to those controlling for co-pollutants.[a]

Pollutant of Interest (IQR)	$PM_{2.5}$ 24-hr avg (11.1 $\mu g/m^3$)	OC 24-hr avg (2.4 $\mu g/m^3$)	EC 24-hr avg (0.42 $\mu g/m^3$)	Nor 24-hr avg (0.43 ng/m^3)	Hop 24-hr avg (0.24 ng/m^3)
**Covariate Pollutant**					
**Fine Particles and Components**					
24-hr avg $PM_{2.5}$	**0.999 (0.981-1.016)**[b]	1.026 (0.999, 1.055)*,[c]	1.019 (1.003, 1.034)**,[d]	1.014 (0.998, 1.031)*	1.015 (1.001, 1.030)**
Major Ions					
24-hr avg SO_4^{2-}	1.003 (0.972, 1.034)	1.020 (1.000, 1.040)**	1.016 (1.002, 1.030)**	1.014 (0.999, 1.029)*	1.013 (1.000, 1.026)**
24-hr avg NO_3^-	0.994 (0.971, 1.017)	1.014 (0.995, 1.034)	1.014 (0.999, 1.029)*	1.013 (0.997, 1.028)	1.011 (0.998, 1.025)*
Carbon					
24-hr avg OC	0.984 (0.957, 1.012)	**1.015 (0.997, 1.033)***,[b]	1.018 (0.998, 1.038)*	1.015 (0.993, 1.037)	1.017 (0.998, 1.037)*
24-hr avg EC	0.993 (0.973, 1.014)	0.997 (0.972, 1.022)	**1.016 (1.002, 1.030)****,[b]	1.019 (0.996, 1.041)	1.016 (0.997, 1.036)*
n-Alkanes					
24-hr avg Oct	0.998 (0.978, 1.018)	1.016 (0.994, 1.038)	1.010 (0.994, 1.027)	1.015 (0.997, 1.032)*	1.016 (1.000, 1.031)**
24-hr avg Non	1.000 (0.980, 1.020)	1.020 (0.998, 1.042)*	1.017 (1.000, 1.033)**	1.018 (1.001, 1.036)**	1.019 (1.003, 1.034)**
Hopanes					
24-hr avg Nor	0.995 (0.975, 1.015)	1.006 (0.981, 1.031)	1.002 (0.983, 1.022)	**1.013 (0.998, 1.028)***,[b]	1.011 (0.985, 1.038)
24-hr avg Hop	0.991 (0.971, 1.011)	0.999 (0.974, 1.026)	1.001 (0.981, 1.022)	1.002 (0.971, 1.033)	**1.012 (1.000, 1.025)***,[b]
PAHs					
24-hr avg Chry	0.992 (0.972, 1.013)	1.015 (0.992, 1.038)	1.010 (0.993, 1.027)	1.017 (0.998, 1.036)*	1.015 (0.999, 1.031)*
24-hr avg BbkF	0.992 (0.972, 1.013)	1.012 (0.989, 1.034)	1.008 (0.990, 1.025)	1.014 (0.995, 1.034)	1.013 (0.997, 1.029)
24-hr avg BaP	0.996 (0.977, 1.017)	1.015 (0.993, 1.037)	1.013 (0.994, 1.032)	1.015 (0.997, 1.033)	1.015 (1.000, 1.030)*
24-hr avg IcdP	0.995 (0.975, 1.016)	1.011 (0.989, 1.033)	1.009 (0.991, 1.028)	1.013 (0.995, 1.031)	1.012 (0.996, 1.028)
Metals and Metalloids					
24-hr avg Si	0.999 (0.981, 1.018)	1.016 (0.996, 1.035)	1.017 (1.002, 1.033)**	1.016 (1.000, 1.032)**	1.014 (1.001, 1.028)**
24-hr avg K	0.999 (0.980, 1.018)	1.015 (0.995, 1.035)	1.015 (1.000, 1.031)**	1.016 (1.000, 1.032)*	1.014 (1.000, 1.028)**
24-hr avg Ca	1.000 (0.980, 1.020)	1.020 (0.998, 1.043)*	1.019 (1.002, 1.036)**	1.017 (1.000, 1.035)**	1.016 (1.001, 1.031)**
24-hr avg Fe	1.005 (0.985, 1.024)	1.018 (0.997, 1.040)*	1.018 (1.000, 1.035)*	1.019 (1.001, 1.037)**	1.016 (1.001, 1.032)**
24-hr avg Cu	0.998 (0.980, 1.017)	1.015 (0.997, 1.034)	1.017 (1.002, 1.032)**	1.016 (1.000, 1.032)*	1.013 (1.000, 1.027)**
24-hr avg Zn	0.995 (0.976, 1.014)	1.012 (0.992, 1.032)	1.014 (0.999, 1.030)*	1.013 (0.996, 1.030)	1.011 (0.997, 1.025)
24-hr avg Pb	0.997 (0.979, 1.016)	1.014 (0.996, 1.033)	1.016 (1.001, 1.032)**	1.015 (0.999, 1.032)*	1.013 (0.999, 1.026)*
**Criteria Pollutants**					
8-hr max O_3	1.004 (0.985, 1.024)	1.019 (1.000, 1.039)**	1.018 (1.003, 1.032)**	1.014 (0.999, 1.029)*	1.013 (1.000, 1.026)*
1-hr max CO	0.991 (0.972, 1.010)	1.009 (0.986, 1.033)	1.011 (0.992, 1.031)	1.009 (0.987, 1.032)	1.010 (0.992, 1.029)
1-hr max NO_2	0.991 (0.970, 1.012)	1.011 (0.988, 1.033)	1.014 (0.998, 1.030)*	1.009 (0.992, 1.026)	1.011 (0.996, 1.026)
1-hr max SO_2	0.996 (0.979, 1.014)	1.014 (0.996, 1.032)	1.015 (1.001, 1.029)**	1.011 (0.996, 1.026)	1.011 (0.998, 1.024)*

[a]Results provided as rate ratios (95% CI) per interquartile range increase in the pollutant of interest indicated in the column heading, controlling for the 'covariate' pollutant indicated at the beginning of the row; Models were 3-day (lags 0-2) distributed lag models with: the 'covariate' pollutant, indicator variables to control for day-of-week, holidays, and to account for one hospital not providing data after April 26, 2002; cubic splines for day of visit with monthly knots; cubic spline for lag 0 maximum temperature with knots placed at the 25th and 75th percentiles; and cubic terms for 1-2 day moving average minimum temperature and 0-2 day moving average dew point temperature; Pollutants of interest for testing in two-pollutant models selected if they had a single-pollutant RR that was equal to or greater than the smallest statistically significant single-pollutant RR greater than 1 for cardiovascular disease (i.e., from results in Table 3 for CVD: RR≥1.012 per IQR, plus $PM_{2.5}$). [b]Refers to the single-pollutant effect (in bold font). [c]Controlling for the non-OC portion of $PM_{2.5}$ produced similar results [RR for OC of 1.023 (95% CI: 1.000, 1.047) per IQR, with 0.05≤p-value<0.10] as controlling for total $PM_{2.5}$. [d]Controlling for the non-EC portion of $PM_{2.5}$ produced similar results [RR for EC of 1.018 (95% CI: 1.003, 1.033) per IQR, with p-value<0.05] as controlling for total $PM_{2.5}$. ** = results with p-value<0.05; * = results with 0.05≤p-value<0.10.

Table S5. Sensitivity analyses of associations between congestive heart failure emergency department visits and ambient pollutants in St. Louis, 6/1/2001-4/30/2003.[a]

Pollutant	IQR	Primary Model[b]	Lag -1 Pollution[c]	2 Knots per Month[d]	1 Knot Every 2 Months[e]	Indicator Lag 0 Maximum Temp[f]	5-Day Distributed Lag[g]
**Fine Particles and Components**							
24-hr avg $PM_{2.5}$	11.1 μg/m^3	1.015 (0.980, 1.051)	1.008 (0.978, 1.039)	1.001 (0.963, 1.039)	1.021 (0.989, 1.055)	1.012 (0.976, 1.049)	1.009 (0.967, 1.054)
Major Ions							
24-hr avg SO_4^{2-}	3.2 μg/m^3	1.008 (0.980, 1.036)	1.012 (0.984, 1.040)	0.997 (0.968, 1.028)	1.012 (0.985, 1.040)	1.005 (0.976, 1.034)	1.003 (0.969, 1.038)
24-hr avg NO_3^-	2.3 μg/m^3	1.007 (0.967, 1.050)	0.998 (0.970, 1.027)	0.986 (0.942, 1.031)	1.012 (0.975, 1.051)	1.006 (0.964, 1.049)	1.013 (0.962, 1.066)
Carbon							
24-hr avg OC	2.4 μg/m^3	1.036 (1.001, 1.072)**	1.006 (0.982, 1.031)	1.032 (0.995, 1.071)*	1.041 (1.007, 1.075)**	1.038 (1.001, 1.076)**	1.056 (1.011, 1.104)**
24-hr avg EC	0.42 μg/m^3	1.042 (1.014, 1.070)**	1.005 (0.984, 1.026)	1.041 (1.011, 1.073)**	1.045 (1.018, 1.073)**	1.044 (1.015, 1.073)**	1.046 (1.008, 1.086)**
n-Alkanes							
24-hr avg Oct	0.77 ng/m^3	1.008 (0.995, 1.020)	1.003 (0.991, 1.015)	1.009 (0.995, 1.024)	1.008 (0.997, 1.020)	1.007 (0.995, 1.020)	1.011 (0.992, 1.029)
24-hr avg Non	1.98 ng/m^3	1.002 (0.985, 1.020)	1.007 (0.994, 1.019)	1.003 (0.982, 1.025)	0.999 (0.984, 1.015)	1.003 (0.985, 1.021)	1.010 (0.988, 1.031)
Hopanes							
24-hr avg Nor	0.43 ng/m^3	1.023 (0.994, 1.052)	1.018 (0.998, 1.038)*	1.026 (0.996, 1.058)*	1.025 (0.998, 1.054)*	1.026 (0.996, 1.056)*	1.033 (0.994, 1.074)
24-hr avg Hop	0.24 ng/m^3	1.023 (0.999, 1.048)*	1.015 (0.999, 1.031)*	1.023 (0.997, 1.050)*	1.025 (1.002, 1.048)**	1.027 (1.001, 1.053)**	1.032 (0.998, 1.066)*
PAHs							
24-hr avg Chry	0.39 ng/m^3	1.013 (0.985, 1.041)	1.012 (0.992, 1.032)	1.012 (0.983, 1.042)	1.018 (0.992, 1.045)	1.017 (0.988, 1.046)	0.997 (0.959, 1.037)
24-hr avg BbkF	0.61 ng/m^3	1.021 (0.995, 1.047)	1.012 (0.993, 1.031)	1.017 (0.989, 1.046)	1.026 (1.002, 1.052)**	1.022 (0.995, 1.050)	1.011 (0.974, 1.048)
24-hr avg BaP	0.19 ng/m^3	1.007 (0.994, 1.020)	1.002 (0.993, 1.011)	1.002 (0.987, 1.017)	1.006 (0.994, 1.018)	1.006 (0.993, 1.020)	1.009 (0.992, 1.027)
24-hr avg IcdP	0.27 ng/m^3	1.018 (0.996, 1.040)	1.011 (0.996, 1.026)	1.014 (0.990, 1.039)	1.021 (1.000, 1.042)**	1.019 (0.997, 1.042)*	1.016 (0.986, 1.046)
Metals and Metalloids							
24-hr avg Si	70.2 ng/m^3	0.998 (0.990, 1.007)	1.005 (0.995, 1.015)	0.999 (0.989, 1.009)	0.997 (0.988, 1.006)	0.999 (0.989, 1.008)	0.993 (0.974, 1.012)
24-hr avg K	35.5 ng/m^3	1.002 (0.983, 1.022)	1.008 (0.988, 1.028)	1.001 (0.981, 1.022)	1.000 (0.982, 1.018)	1.001 (0.982, 1.021)	1.008 (0.980, 1.036)
24-hr avg Ca	86.0 ng/m^3	1.021 (0.993, 1.050)	1.010 (0.992, 1.028)	1.022 (0.992, 1.054)	1.014 (0.987, 1.040)	1.021 (0.992, 1.050)	1.021 (0.986, 1.058)
24-hr avg Fe	85.9 ng/m^3	1.006 (0.984, 1.029)	1.017 (0.999, 1.036)*	1.001 (0.977, 1.026)	1.003 (0.982, 1.025)	1.006 (0.983, 1.030)	1.002 (0.971, 1.034)
24-hr avg Cu	21.9 ng/m^3	0.994 (0.980, 1.008)	1.000 (0.992, 1.008)	0.992 (0.977, 1.006)	0.992 (0.979, 1.006)	0.991 (0.977, 1.005)	0.996 (0.978, 1.015)
24-hr avg Zn	37.9 ng/m^3	1.017 (1.003, 1.031)**	0.999 (0.991, 1.008)	1.012 (0.998, 1.027)	1.015 (1.001, 1.028)**	1.016 (1.001, 1.031)**	1.015 (0.996, 1.035)
24-hr avg Pb	14.1 ng/m^3	0.997 (0.985, 1.009)	1.004 (0.998, 1.010)	0.993 (0.979, 1.007)	0.998 (0.986, 1.010)	0.994 (0.982, 1.007)	1.003 (0.986, 1.020)
**Criteria Gases**							
8-hr max O_3	28.3 ppb	1.057 (0.982, 1.139)	1.045 (0.998, 1.093)*	1.052 (0.970, 1.140)	1.061 (0.989, 1.138)	1.056 (0.978, 1.140)	1.065 (0.978, 1.160)
1-hr max CO	0.7 ppm	1.015 (0.989, 1.041)	1.015 (0.997, 1.033)	1.011 (0.984, 1.040)	1.023 (0.998, 1.048)*	1.014 (0.988, 1.041)	1.025 (0.993, 1.059)
1-hr max NO_2	12.0 ppb	1.011 (0.973, 1.050)	1.014 (0.989, 1.041)	1.005 (0.965, 1.048)	1.024 (0.990, 1.059)	1.012 (0.973, 1.053)	1.029 (0.982, 1.077)
1-hr max SO_2	24.0 ppb	1.006 (0.988, 1.024)	1.005 (0.994, 1.016)	1.001 (0.981, 1.022)	1.007 (0.990, 1.025)	1.001 (0.982, 1.019)	1.003 (0.980, 1.027)

[a]Results provided as rate ratios (95% CI) per interquartile range increase in pollution. [b]"Primary Model" was a 3-day (lags 0-2) distributed lag model with: indicator variables to control for day-of-week, holidays, and to account for one hospital not providing data after April 26, 2002; cubic splines for day of visit with monthly knots; cubic spline for lag 0 maximum temperature with knots placed at the 25th and 75th percentiles; and cubic terms for 1-2 day moving average minimum temperature and 0-2 day moving average dew point temperature. [c]"Lag -1 Pollution" model was the same as the primary model, but included pollutant concentrations on the day after the emergency department visit (lag -1) given pollutant levels on the days of interest (results for lag -1 RRs reported here). [d]"2 Knots per Month" model was the same as the primary model, but included a cubic spline for day of visit with two knots instead of one knot per month. [e]"1 Knot Every 2 Months" model was the same as the primary model, but included a cubic spline for day of visit with one knot every two months instead of one knot every month. [f]"Indicator Lag 0 Max Temp" model was the same as the primary model, but included indicator variables for each degree Celsius of lag 0 max temp instead of a cubic spline. [g]"5-Day Distributed Lag" model was similar to the primary model, but examined pollutant lags 0-4 with control for minimum and dew point temperature adjusted to include the moving average of lags 1-4 and 0-4 respectively. ** = results with p-value<0.05; * = results with 0.05≤p-value<0.10.

Table S6. Comparison of selected single-pollutant model results for congestive heart failure to those controlling for co-pollutants.[a]

Pollutant of Interest (IQR)	$PM_{2.5}$ 24-hr avg (11.1 µg/m^3)	OC 24-hr avg (2.4 µg/m^3)	EC 24-hr avg (0.42 µg/m^3)	Nor 24-hr avg (0.43 ng/m^3)	Hop 24-hr avg (0.24 ng/m^3)	BbkF 24-hr avg (0.61 ng/m^3)	IcdP 24-hr avg (0.27 ng/m^3)	Ca 24-hr avg (86.0 ng/m^3)	Zn 24-hr avg (37.9 ng/m^3)	O_3 8-hr max (28.3 ppb)
**Covariate Pollutant**										
**Fine Particles and Components**										
24-hr avg $PM_{2.5}$	**1.015 (0.980, 1.051)**[b]	1.059 (1.005,¸ 1.117)**,[c]	1.049 (1.018, 1.080)**,[d]	1.026 (0.994, 1.059)	1.028 (1.001, 1.057)**	1.026 (0.996, 1.057)*	1.020 (0.996, 1.044)	1.017 (0.986, 1.050)	1.016 (1.002, 1.031)**	1.031 (0.951, 1.118)
Major Ions										
24-hr avg SO_4^{2-}	1.033 (0.972, 1.097)	1.042 (1.004, 1.082)**	1.042 (1.014, 1.071)**	1.024 (0.995, 1.053)	1.024 (0.999, 1.049)*	1.022 (0.995, 1.049)	1.019 (0.997, 1.041)*	1.021 (0.993, 1.050)	1.017 (1.003, 1.031)**	1.054 (0.966, 1.149)
24-hr avg NO_3^-	1.024 (0.979, 1.072)	1.046 (1.007, 1.087)**	1.049 (1.020, 1.080)**	1.031 (1.000, 1.063)**	1.028 (1.003, 1.055)**	1.021 (0.992, 1.051)	1.019 (0.994, 1.044)	1.023 (0.995, 1.053)	1.017 (1.003, 1.032)**	1.047 (0.968, 1.131)
Carbon										
24-hr avg OC	0.973 (0.920, 1.029)	**1.036 (1.001, 1.072)**[b,**]	1.048 (1.008, 1.089)**	1.006 (0.965, 1.049)	1.015 (0.977, 1.054)	1.012 (0.977, 1.047)	1.003 (0.973, 1.034)	1.017 (0.980, 1.055)	1.012 (0.997, 1.028)	1.025 (0.947, 1.111)
24-hr avg EC	0.990 (0.950, 1.031)	0.990 (0.942, 1.041)	**1.042 (1.014, 1.070)**[b,**]	1.000 (0.958, 1.044)	1.001 (0.964, 1.040)	1.011 (0.976, 1.047)	0.996 (0.964, 1.028)	1.011 (0.976, 1.047)	1.009 (0.994, 1.025)	1.036 (0.958, 1.120)
n-Alkanes										
24-hr avg Oct	0.999 (0.961, 1.039)	1.031 (0.988, 1.076)	1.032 (1.000, 1.065)*	1.014 (0.981, 1.049)	1.019 (0.990, 1.049)	1.009 (0.978, 1.041)	1.007 (0.982, 1.032)	1.025 (0.991, 1.059)	1.014 (1.000, 1.029)*	1.041 (0.962, 1.128)
24-hr avg Non	1.009 (0.971, 1.048)	1.047 (1.004, 1.093)**	1.046 (1.013, 1.080)**	1.026 (0.993, 1.06)	1.029 (1.000, 1.059)*	1.026 (0.996, 1.057)*	1.019 (0.994, 1.044)	1.036 (1.002, 1.071)**	1.017 (1.003, 1.032)**	1.032 (0.955, 1.116)
Hopanes										
24-hr avg Nor	1.001 (0.962, 1.040)	1.035 (0.986, 1.086)	1.041 (1.001, 1.082)**	**1.023 (0.994, 1.052)**[b]	1.029 (0.977, 1.083)	1.013 (0.980, 1.047)	1.009 (0.983, 1.036)	1.029 (0.995, 1.063)*	1.014 (0.999, 1.029)*	1.036 (0.957, 1.122)
24-hr avg Hop	0.995 (0.957, 1.036)	1.024 (0.973, 1.077)	1.040 (0.999, 1.082)*	0.993 (0.935, 1.055)	**1.023 (0.999, 1.048)**[b,*]	1.011 (0.979, 1.043)	1.008 (0.981, 1.035)	1.025 (0.990, 1.060)	1.013 (0.999, 1.028)*	1.036 (0.958, 1.120)
PAHs										
24-hr avg Chry	1.007 (0.966, 1.048)	1.043 (0.999, 1.090)*	1.041 (1.007, 1.076)**	1.023 (0.987, 1.061)	1.026 (0.996, 1.057)*	1.061 (0.993, 1.134)*	1.022 (0.993, 1.053)	1.031 (0.998, 1.064)*	1.016 (1.001, 1.031)**	1.052 (0.974, 1.137)
24-hr avg BbkF	0.998 (0.959, 1.040)	1.028 (0.985, 1.074)	1.033 (0.998, 1.069)*	1.015 (0.978, 1.053)	1.019 (0.988, 1.050)	**1.021 (0.995, 1.047)**[b]	1.014 (0.980, 1.049)	1.027 (0.995, 1.060)	1.015 (1.000, 1.030)**	1.057 (0.978, 1.141)
24-hr avg BaP	1.006 (0.967, 1.047)	1.033 (0.990, 1.078)	1.042 (1.005, 1.081)**	1.021 (0.986, 1.056)	1.023 (0.994, 1.053)	1.024 (0.979, 1.072)	1.019 (0.988, 1.052)	1.031 (0.998, 1.065)*	1.017 (1.002, 1.033)**	1.053 (0.974, 1.139)
24-hr avg IcdP	1.007 (0.967, 1.048)	1.036 (0.993, 1.081)	1.043 (1.007, 1.081)**	1.017 (0.983, 1.054)	1.018 (0.988, 1.049)	1.007 (0.966, 1.049)	**1.018 (0.996, 1.040)**[b]	1.027 (0.994, 1.061)	1.016 (1.001, 1.031)**	1.050 (0.971, 1.136)
Metals and Metalloids										
24-hr avg Si	1.021 (0.985, 1.059)	1.031 (0.994, 1.070)	1.042 (1.012, 1.072)**	1.023 (0.993, 1.054)	1.022 (0.997, 1.048)*	1.018 (0.991, 1.046)	1.019 (0.994, 1.045)	1.035 (1.002, 1.068)**	1.017 (1.003, 1.032)**	1.052 (0.973, 1.137)
24-hr avg K	1.017 (0.980, 1.057)	1.035 (0.996, 1.076)*	1.048 (1.018, 1.079)**	1.025 (0.994, 1.057)	1.025 (0.999, 1.052)*	1.019 (0.991, 1.048)	1.020 (0.994, 1.046)	1.024 (0.992, 1.057)	1.017 (1.003, 1.032)**	1.045 (0.967, 1.129)
24-hr avg Ca	1.012 (0.974, 1.053)	1.029 (0.985, 1.074)	1.043 (1.010, 1.077)**	1.018 (0.985, 1.051)	1.019 (0.991, 1.048)	1.016 (0.987, 1.045)	1.016 (0.990, 1.043)	**1.021 (0.993, 1.050)**[b]	1.015 (1.001, 1.030)**	1.041 (0.962, 1.125)
24-hr avg Fe	1.023 (0.984, 1.062)	1.026 (0.984, 1.069)	1.040 (1.005, 1.076)**	1.015 (0.981, 1.050)	1.018 (0.989, 1.047)	1.010 (0.978, 1.044)	1.015 (0.986, 1.046)	1.028 (0.991, 1.066)	1.017 (1.003, 1.032)**	1.054 (0.976, 1.138)
24-hr avg Cu	1.019 (0.984, 1.056)	1.041 (1.005, 1.079)**	1.050 (1.021, 1.079)**	1.030 (1.000, 1.061)*	1.028 (1.003, 1.054)**	1.023 (0.996, 1.051)*	1.023 (0.998, 1.049)*	1.021 (0.993, 1.050)	1.019 (1.005, 1.034)**	1.051 (0.973, 1.135)
24-hr avg Zn	1.011 (0.975, 1.049)	1.029 (0.991, 1.069)	1.042 (1.011, 1.073)**	1.017 (0.985, 1.050)	1.021 (0.995, 1.047)	1.015 (0.988, 1.044)	1.017 (0.992, 1.044)	1.012 (0.984, 1.042)	**1.017 (1.003, 1.031)**[b,**]	1.045 (0.968, 1.128)
24-hr avg Pb	1.022 (0.986, 1.060)	1.043 (1.006, 1.081)**	1.055 (1.025, 1.086)**	1.031 (1.000, 1.064)*	1.029 (1.003, 1.056)**	1.026 (0.998, 1.055)*	1.026 (1.000, 1.053)*	1.023 (0.995, 1.053)	1.023 (1.007, 1.038)**	1.053 (0.976, 1.137)
**Criteria Gases**										
8-hr max O_3	1.015 (0.977, 1.054)	1.042 (1.004, 1.080)**	1.048 (1.020, 1.078)**	1.025 (0.996, 1.055)*	1.025 (0.999, 1.050)*	1.023 (0.997, 1.050)*	1.019 (0.998, 1.041)*	1.023 (0.994, 1.054)	1.019 (1.005, 1.033)**	**1.057 (0.982, 1.139)**[b]
1-hr max CO	1.005 (0.968, 1.043)	1.024 (0.979, 1.071)	1.036 (0.999, 1.075)*	0.999 (0.958, 1.042)	1.009 (0.975, 1.045)	1.009 (0.975, 1.043)	1.010 (0.982, 1.039)	1.013 (0.982, 1.045)	1.001 (0.977, 1.024)	1.085 (1.006, 1.171)**
1-hr max NO_2	1.015 (0.973, 1.058)	1.054 (1.009, 1.100)**	1.049 (1.018, 1.081)**	1.021 (0.989, 1.054)	1.022 (0.993, 1.051)	1.019 (0.992, 1.048)	1.018 (0.995, 1.042)	1.044 (1.008, 1.081)**	1.017 (1.002, 1.031)**	1.022 (0.941, 1.112)
1-hr max SO_2	1.014 (0.979, 1.051)	1.036 (1.001, 1.073)**	1.042 (1.014, 1.070)**	1.022 (0.993, 1.052)	1.022 (0.997, 1.047)*	1.020 (0.994, 1.047)	1.018 (0.996, 1.040)	1.018 (0.989, 1.047)	1.017 (1.003, 1.032)**	1.057 (0.981, 1.139)

[a]Results provided as rate ratios (95% CI) per interquartile range increase in the pollutant of interest indicated in the column heading, controlling for the 'covariate' pollutant indicated at the beginning of the row; Models were 3-day (lags 0-2) distributed lag models with: the 'covariate' pollutant, indicator variables to control for day-of-week, holidays, and to account for one hospital not providing data after April 26, 2002; cubic splines for day of visit with monthly knots; cubic spline for lag 0 maximum temperature with knots placed at the 25th and 75th percentiles; and cubic terms for 1-2 day moving average minimum temperature and 0-2 day moving average dew point temperature; Pollutants of interest for testing in two-pollutant models selected if they had a single-pollutant RR that was equal to or greater than the smallest statistically significant single-pollutant RR greater than 1 for congestive heart failure (i.e., from results in Table 3 for CHF: RR≥1.017 per IQR, plus $PM_{2.5}$).
[b]Refers to the single-pollutant effect (in bold font). [c]Controlling for the non-OC portion of $PM_{2.5}$ produced similar results [RR for OC of 1.053 (95% CI: 1.007, 1.101) per IQR, with p-value<0.05] as controlling for total $PM_{2.5}$. [d]Controlling for the non-EC portion of $PM_{2.5}$ produced similar results [RR for EC of 1.048 (95% CI: 1.018, 1.079) per IQR, with p-value<0.05] as controlling for total $PM_{2.5}$. ** = results with p-value<0.05; * = results with 0.05≤p-value<0.10.

Table S7. Sensitivity analyses of associations between asthma/wheeze emergency department visits and ambient pollutants in St. Louis, 6/1/2001-4/30/2003.[a]

Pollutant	IQR	Primary Model[b]	Lag -1 Pollution[c]	2 Knots per Month[d]	1 Knot Every 2 Months[e]	Indicator Lag 0 Maximum Temp[f]	5-Day Distributed Lag[g]
**Fine Particles and Components**							
24-hr avg $PM_{2.5}$	11.1 μg/m^3	1.040 (1.009, 1.071)**	1.022 (0.996, 1.048)*	1.017 (0.986, 1.050)	1.030 (1.002, 1.060)**	1.037 (1.006, 1.069)**	1.047 (1.008, 1.087)**
Major Ions							
24-hr avg SO_4^{2-}	3.2 μg/m^3	1.029 (1.004, 1.055)**	1.019 (0.995, 1.043)	1.015 (0.990, 1.041)	1.024 (1.000, 1.049)*	1.029 (1.003, 1.055)**	1.033 (1.001, 1.067)**
24-hr avg NO_3^-	2.3 μg/m^3	1.011 (0.977, 1.046)	1.020 (0.996, 1.045)*	0.991 (0.956, 1.028)	1.003 (0.971, 1.035)	1.010 (0.975, 1.046)	1.013 (0.970, 1.057)
Carbon							
24-hr avg OC	2.4 μg/m^3	1.029 (1.000, 1.060)*	1.010 (0.989, 1.031)	1.015 (0.985, 1.046)	1.018 (0.990, 1.047)	1.032 (1.001, 1.064)**	1.044 (1.005, 1.084)**
24-hr avg EC	0.42 μg/m^3	1.020 (0.998, 1.044)*	1.011 (0.994, 1.028)	1.005 (0.981, 1.030)	1.015 (0.992, 1.038)	1.020 (0.996, 1.045)	1.043 (1.010, 1.077)**
n-Alkanes							
24-hr avg Oct	0.77 ng/m^3	1.003 (0.993, 1.013)	1.000 (0.991, 1.009)	1.001 (0.989, 1.013)	1.003 (0.993, 1.012)	1.004 (0.994, 1.015)	0.998 (0.983, 1.013)
24-hr avg Non	1.98 ng/m^3	1.003 (0.989, 1.018)	0.997 (0.987, 1.008)	1.001 (0.984, 1.018)	1.002 (0.989, 1.015)	1.005 (0.990, 1.020)	1.002 (0.984, 1.021)
Hopanes							
24-hr avg Nor	0.43 ng/m^3	1.011 (0.988, 1.036)	0.998 (0.982, 1.014)	1.002 (0.978, 1.027)	1.004 (0.981, 1.027)	1.011 (0.986, 1.036)	1.018 (0.986, 1.052)
24-hr avg Hop	0.24 ng/m^3	1.027 (1.006, 1.047)**	1.000 (0.987, 1.014)	1.012 (0.991, 1.033)	1.019 (1.000, 1.039)**	1.031 (1.009, 1.052)**	1.036 (1.008, 1.065)**
PAHs							
24-hr avg Chry	0.39 ng/m^3	1.018 (0.995, 1.042)	1.009 (0.992, 1.026)	1.012 (0.988, 1.037)	1.020 (0.998, 1.043)*	1.019 (0.995, 1.045)	1.016 (0.984, 1.050)
24-hr avg BbkF	0.61 ng/m^3	1.017 (0.996, 1.039)	1.008 (0.993, 1.024)	1.010 (0.987, 1.032)	1.018 (0.998, 1.040)*	1.016 (0.994, 1.039)	1.014 (0.984, 1.045)
24-hr avg BaP	0.19 ng/m^3	1.006 (0.996, 1.017)	1.004 (0.997, 1.010)	0.998 (0.987, 1.010)	1.006 (0.996, 1.016)	1.005 (0.994, 1.016)	1.010 (0.996, 1.024)
24-hr avg IcdP	0.27 ng/m^3	1.013 (0.996, 1.031)	1.009 (0.997, 1.021)	1.001 (0.982, 1.020)	1.011 (0.994, 1.029)	1.013 (0.995, 1.032)	1.028 (1.004, 1.054)**
Metals and Metalloids							
24-hr avg Si	70.2 ng/m^3	1.002 (0.994, 1.010)	1.007 (0.998, 1.016)	1.002 (0.993, 1.010)	1.001 (0.993, 1.009)	1.003 (0.995, 1.011)	1.007 (0.991, 1.024)
24-hr avg K	35.5 ng/m^3	1.012 (0.994, 1.029)	0.995 (0.978, 1.012)	1.008 (0.989, 1.026)	1.007 (0.991, 1.024)	1.011 (0.993, 1.029)	1.027 (1.003, 1.053)**
24-hr avg Ca	86.0 ng/m^3	1.024 (1.001, 1.048)**	1.011 (0.996, 1.027)	1.012 (0.988, 1.038)	1.021 (0.998, 1.044)*	1.020 (0.996, 1.046)	1.053 (1.022, 1.085)**
24-hr avg Fe	85.9 ng/m^3	1.014 (0.994, 1.034)	1.008 (0.993, 1.024)	1.004 (0.983, 1.025)	1.011 (0.992, 1.030)	1.014 (0.993, 1.034)	1.044 (1.017, 1.072)**
24-hr avg Cu	21.9 ng/m^3	1.000 (0.989, 1.011)	0.993 (0.987, 1.000)**	1.006 (0.994, 1.017)	0.998 (0.987, 1.009)	0.998 (0.987, 1.010)	1.001 (0.987, 1.016)
24-hr avg Zn	37.9 ng/m^3	0.993 (0.981, 1.006)	0.999 (0.992, 1.007)	0.988 (0.975, 1.001)*	0.992 (0.980, 1.004)	0.992 (0.980, 1.005)	1.000 (0.984, 1.016)
24-hr avg Pb	14.1 ng/m^3	1.002 (0.992, 1.013)	1.001 (0.996, 1.007)	0.998 (0.987, 1.010)	1.002 (0.992, 1.012)	1.003 (0.992, 1.013)	1.005 (0.991, 1.019)
**Criteria Gases**							
8-hr max O_3	28.3 ppb	1.067 (1.001, 1.137)**	0.978 (0.941, 1.016)	1.060 (0.992, 1.133)*	1.066 (1.003, 1.133)**	1.073 (1.005, 1.146)**	1.104 (1.027, 1.188)**
1-hr max CO	0.7 ppm	1.015 (0.993, 1.036)	1.008 (0.993, 1.023)	1.011 (0.989, 1.034)	1.013 (0.993, 1.034)	1.016 (0.994, 1.039)	1.021 (0.993, 1.049)
1-hr max NO_2	12.0 ppb	1.050 (1.018, 1.084)**	1.010 (0.988, 1.032)	1.037 (1.003, 1.071)**	1.035 (1.005, 1.065)**	1.048 (1.015, 1.083)**	1.081 (1.040, 1.123)**
1-hr max SO_2	24.0 ppb	0.996 (0.981, 1.011)	1.002 (0.992, 1.011)	0.993 (0.977, 1.010)	1.000 (0.985, 1.015)	0.997 (0.982, 1.013)	1.004 (0.985, 1.024)

[a]Results provided as rate ratios (95% CI) per interquartile range increase in pollution. [b]"Primary Model" was a 3-day (lags 0-2) distributed lag model with: indicator variables to control for season, day-of-week, holidays, and to account for one hospital not providing data after April 26, 2002; cubic splines for day of visit with monthly knots; cubic spline for lag 0 maximum temperature with knots placed at the 25th and 75th percentiles; and cubic terms for 1-2 day moving average minimum temperature and 0-2 day moving average dew point temperature. [c]"Lag -1 Pollution" model was the same as the primary model, but included pollutant concentrations on the day after the emergency department visit (lag -1) given pollutant levels on the days of interest (results for lag -1 RRs reported here). [d]"2 Knots per Month" model was the same as the primary model, but included a cubic spline for day of visit with two knots instead of one knot per month. [e]"1 Knot Every 2 Months" model was the same as the primary model, but included a cubic spline for day of visit with one knot every two months instead of one knot every month. [f]"Indicator Lag 0 Max Temp" model was the same as the primary model, but included indicator variables for each degree Celsius of lag 0 max temp instead of a cubic spline. [g]"5-Day Distributed Lag" model was similar to the primary model, but examined pollutant lags 0-4 with control for minimum and dew point temperature adjusted to include the moving average of lags 1-4 and 0-4 respectively. ** = results with p-value<0.05; * = results with 0.05≤p-value<0.10.

Table S8. Comparison of selected single-pollutant model results for asthma/wheeze to those controlling for co-pollutants.[a]

Pollutant of Interest (IQR)	$PM_{2.5}$ 24-hr avg (11.1 μg/m^3)	SO_4^{2-} 24-hr avg (3.2 μg/m^3)	OC 24-hr avg (2.4 μg/m^3)	EC 24-hr avg (0.42 μg/m^3)	Hop 24-hr avg (0.24 ng/m^3)	Ca 24-hr avg (86.0 ng/m^3)	O_3 8-hr max (28.3 ppb)	NO_2 1-hr max (12.0 ppb)
**Covariate Pollutant**								
**Fine Particles and Components**								
24-hr avg $PM_{2.5}$	**1.040 (1.009, 1.071)**[b,**]	1.001 (0.959, 1.046)[c]	1.011 (0.966, 1.059)[d]	1.013 (0.987, 1.040)[e]	1.022 (0.999, 1.045)*	1.023 (0.996, 1.050)*	1.058 (0.987, 1.135)	1.052 (1.012, 1.092)**
Major Ions								
24-hr avg SO_4^{2-}	1.040 (0.987, 1.095)	**1.029 (1.004, 1.055)**[b,**]	1.021 (0.989, 1.054)	1.019 (0.996, 1.043)	1.025 (1.005, 1.046)**	1.023 (0.999, 1.048)*	1.053 (0.979, 1.133)	1.046 (1.011, 1.082)**
24-hr avg NO_3^-	1.061 (1.020, 1.103)**	1.031 (1.003, 1.060)**	1.030 (0.997, 1.063)*	1.018 (0.993, 1.043)	1.026 (1.005, 1.048)**	1.020 (0.996, 1.045)	1.055 (0.987, 1.127)	1.052 (1.016, 1.088)**
Carbon								
24-hr avg OC	1.030 (0.981, 1.083)	1.023 (0.994, 1.052)	**1.029 (1.000, 1.060)**[b,*]	1.010 (0.977, 1.044)	1.019 (0.987, 1.051)	1.020 (0.989, 1.052)	1.075 (1.003, 1.152)**	1.058 (1.015, 1.103)**
24-hr avg EC	1.029 (0.992, 1.067)	1.028 (1.001, 1.055)**	1.019 (0.975, 1.064)	**1.020 (0.998, 1.044)**[b,*]	1.028 (0.996, 1.061)*	1.022 (0.992, 1.054)	1.077 (1.006, 1.154)**	1.062 (1.022, 1.103)**
n-Alkanes								
24-hr avg Oct	1.044 (1.010, 1.079)**	1.033 (1.006, 1.060)**	1.038 (1.003, 1.075)**	1.033 (1.006, 1.060)**	1.035 (1.010, 1.060)**	1.030 (1.002, 1.058)**	1.071 (1.001, 1.146)**	1.055 (1.020, 1.092)**
24-hr avg Non	1.045 (1.011, 1.080)**	1.033 (1.006, 1.060)**	1.047 (1.010, 1.084)**	1.033 (1.006, 1.060)**	1.035 (1.010, 1.060)**	1.033 (1.005, 1.061)**	1.077 (1.008, 1.151)**	1.059 (1.024, 1.096)**
Hopanes								
24-hr avg Nor	1.039 (1.004, 1.074)**	1.030 (1.004, 1.057)**	1.048 (1.006, 1.091)**	1.033 (1.000, 1.067)*	1.086 (1.041, 1.134)**	1.031 (1.003, 1.060)**	1.090 (1.018, 1.166)**	1.061 (1.023, 1.099)**
24-hr avg Hop	1.027 (0.993, 1.063)	1.030 (1.003, 1.056)**	1.013 (0.971, 1.058)	1.005 (0.972, 1.039)	**1.027 (1.006, 1.047)**[b,**]	1.019 (0.990, 1.048)	1.080 (1.011, 1.155)**	1.045 (1.008, 1.083)**
PAHs								
24-hr avg Chry	1.040 (1.005, 1.077)**	1.032 (1.006, 1.059)**	1.034 (0.997, 1.073)*	1.025 (0.997, 1.054)*	1.027 (1.002, 1.053)**	1.025 (0.998, 1.053)*	1.076 (1.008, 1.148)**	1.054 (1.018, 1.092)**
24-hr avg BbkF	1.038 (1.003, 1.074)**	1.030 (1.003, 1.056)**	1.033 (0.997, 1.071)*	1.028 (0.999, 1.058)*	1.026 (1.001, 1.052)**	1.026 (0.999, 1.053)*	1.078 (1.010, 1.150)**	1.053 (1.018, 1.090)**
24-hr avg BaP	1.036 (1.002, 1.071)**	1.031 (1.005, 1.058)**	1.032 (0.996, 1.069)*	1.033 (1.003, 1.064)**	1.026 (1.002, 1.051)**	1.022 (0.995, 1.050)	1.078 (1.010, 1.152)**	1.052 (1.017, 1.089)**
24-hr avg IcdP	1.036 (1.002, 1.072)**	1.027 (1.000, 1.053)**	1.032 (0.997, 1.069)*	1.028 (0.999, 1.058)*	1.028 (1.003, 1.054)**	1.023 (0.996, 1.051)*	1.081 (1.013, 1.155)**	1.054 (1.017, 1.091)**
Metals and Metalloids								
24-hr avg Si	1.039 (1.007, 1.072)**	1.030 (1.003, 1.057)**	1.013 (0.982, 1.046)	1.011 (0.986, 1.036)	1.021 (0.999, 1.042)*	1.027 (1.000, 1.055)*	1.065 (0.996, 1.139)*	1.040 (1.003, 1.078)**
24-hr avg K	1.035 (1.001, 1.070)**	1.026 (1.000, 1.053)**	1.015 (0.981, 1.049)	1.013 (0.988, 1.039)	1.020 (0.998, 1.043)*	1.020 (0.993, 1.048)	1.063 (0.994, 1.137)*	1.045 (1.008, 1.083)**
24-hr avg Ca	1.027 (0.993, 1.062)	1.025 (0.999, 1.052)*	1.010 (0.974, 1.048)	1.008 (0.980, 1.036)	1.020 (0.996, 1.044)	**1.024 (1.001, 1.048)**[b,**]	1.059 (0.989, 1.133)*	1.047 (1.006, 1.089)**
24-hr avg Fe	1.034 (1.000, 1.070)*	1.027 (1.001, 1.053)**	1.019 (0.982, 1.056)	1.011 (0.982, 1.042)	1.023 (0.998, 1.048)*	1.025 (0.994, 1.058)	1.066 (0.998, 1.140)*	1.048 (1.007, 1.089)**
24-hr avg Cu	1.041 (1.009, 1.073)**	1.029 (1.004, 1.056)**	1.026 (0.995, 1.058)*	1.019 (0.995, 1.043)	1.028 (1.007, 1.049)**	1.024 (1.000, 1.048)**	1.070 (1.001, 1.143)**	1.051 (1.016, 1.087)**
24-hr avg Zn	1.049 (1.016, 1.083)**	1.030 (1.004, 1.057)**	1.038 (1.005, 1.072)**	1.027 (1.001, 1.053)**	1.033 (1.011, 1.056)**	1.031 (1.006, 1.057)**	1.073 (1.004, 1.147)**	1.056 (1.020, 1.093)**
24-hr avg Pb	1.041 (1.009, 1.074)**	1.029 (1.004, 1.056)**	1.028 (0.997, 1.060)*	1.023 (0.998, 1.049)*	1.026 (1.005, 1.048)**	1.024 (1.000, 1.049)*	1.070 (1.002, 1.143)**	1.050 (1.015, 1.087)**
**Criteria Gases**								
8-hr max O_3	1.021 (0.988, 1.054)	1.014 (0.986, 1.043)	1.009 (0.977, 1.041)	1.012 (0.988, 1.036)	1.023 (1.002, 1.044)**	1.015 (0.989, 1.042)	**1.067 (1.001, 1.137)**[b,**]	1.040 (1.003, 1.079)**
1-hr max CO	1.033 (1.000, 1.067)**	1.027 (1.001, 1.052)**	1.038 (0.999, 1.079)*	1.025 (0.993, 1.057)	1.040 (1.010, 1.070)**	1.018 (0.992, 1.046)	1.053 (0.986, 1.125)	1.047 (1.011, 1.085)**
1-hr max NO_2	1.009 (0.974, 1.045)	1.015 (0.988, 1.043)	0.994 (0.958, 1.030)	1.000 (0.975, 1.026)	1.015 (0.992, 1.039)	1.004 (0.975, 1.034)	1.036 (0.964, 1.114)	**1.050 (1.018, 1.084)**[b,**]
1-hr max SO_2	1.041 (1.010, 1.073)**	1.030 (1.005, 1.056)**	1.030 (1.000, 1.060)*	1.021 (0.998, 1.044)*	1.027 (1.007, 1.048)**	1.026 (1.001, 1.051)**	1.068 (1.001, 1.139)**	1.052 (1.019, 1.086)**

[a]Results provided as rate ratios (95% CI) per interquartile range increase in the pollutant of interest indicated in the column heading, controlling for the 'covariate' pollutant indicated at the beginning of the row; Models were 3-day (lags 0-2) distributed lag models with: the 'covariate' pollutant, indicator variables to control for season, day-of-week, holidays, and to account for one hospital not providing data after April 26, 2002; cubic splines for day of visit with monthly knots; cubic spline for lag 0 maximum temperature with knots placed at the 25th and 75th percentiles; and cubic terms for 1-2 day moving average minimum temperature and 0-2 day moving average dew point temperature; Pollutants of interest for testing in two-pollutant models selected if they had a single-pollutant RR that was equal to or greater than the smallest statistically significant single-pollutant RR greater than 1 for asthma/wheeze (i.e., from results in Table 4 for asthma/wheeze: RR≥1.020 per IQR). [b]Refers to the single-pollutant effect (in bold font). [c]Controlling for the non-SO_4^{2-} portion of $PM_{2.5}$ produced a slightly higher RR [RR for SO_4^{2-} of 1.017 (95% CI: 0.987, 1.047) per IQR] than controlling for total $PM_{2.5}$. [d]Controlling for the non-OC portion of $PM_{2.5}$ produced similar results [RR for OC of 1.017 (95% CI: 0.979, 1.058) per IQR] as controlling for total $PM_{2.5}$. [e]Controlling for the non-EC portion of $PM_{2.5}$ produced similar results [RR for EC of 1.015 (95% CI: 0.989, 1.041) per IQR] as controlling for total $PM_{2.5}$. ** = results with p-value<0.05; * = results with 0.05≤p-value<0.10.
